# Comparative phenotypic, physiological, and transcriptomic responses to drought and recovery in two *Fraxinus* species

**DOI:** 10.1186/s12870-025-06372-6

**Published:** 2025-03-18

**Authors:** Tae-Lim Kim, Hyemin Lim, Kyungmi Lee, Michael Immanuel Jesse Denison, Sathishkumar Natarajan, Changyoung Oh

**Affiliations:** 1 Department of Forest Bioresources, National Institute of Forest Science, Suwon, 16631 Korea; 2 3BIGS CO. Ltd, Hwaseong, 18469 Republic of Korea

**Keywords:** Fraxinus chiisanensis_1_, Fraxinus rhynchophylla_2_, transcriptome_3_, Physiological response_4_, Drought stress_5_

## Abstract

**Background:**

This study focused on the drought tolerance and resilience of two ash species: *Fraxinus chiisanensis* and *F. rhynchophylla*. These two species are distributed in different habitats, suggesting that they have different levels of drought tolerance. Understanding their response to drought stress, particularly during the seedling stage, is crucial for selecting and developing drought-resistant varieties. This study aimed to compare the phenotypic, physiological, and transcriptomic characteristics of drought-stressed and recovered rewatered plants in a time-course experiment.

**Results:**

In *F. rhynchophylla*, drought stress resulted in more severe growth retardation, temperature increase, and a faster decline in the fluorescence response, accompanied by a significant rise in stress indices. However, these reactions recovered quickly after rehydration. In contrast, *F. chiisanensis* exhibited less growth retardation, a slower decline in fluorescence, and milder increases in stress indices, although many individuals did not fully recover after rehydration. The activity of antioxidant enzymes (SOD, CAT, APX) was more responsive and recovered more efficiently in *F. rhynchophylla*, while *F. chiisanensis* had a weaker and delayed response. Transcriptome analysis revealed that photosynthesis and enzyme activity were the most responsive to drought and recovery, as shown by Gene Ontology term analysis. Kyoto Encyclopedia of Genes and Genomes pathway analysis identified common pathways involved in starch and sucrose metabolism and phenylpropanoid biosynthesis in both species. *F. rhynchophylla* had more differentially expressed genes (DEGs) than *F. chiisanensis*, particularly on the drought and recovery day 6. Most drought-induced DEGs were restored after rehydration. Commonly associated genes included *BGLU* and *TPS* in sugar metabolism; *CAT*, *GSTF*, *TT7*, and *HCT* in antioxidant enzymes; *PYL4* and *RR17* in hormone signaling; and *ADC1* and *ASP3* in proline synthesis.

**Conclusions:**

This study highlights the species-specific characteristics of drought and recovery responses of two *Fraxinus* species and provides targets for assessing and improving drought tolerance. Moreover, the results of this study provide insights into the physiological and genetic responses of *Fraxinus* and may guide future research on ash tree stress tolerance.

**Supplementary Information:**

The online version contains supplementary material available at 10.1186/s12870-025-06372-6.

## Background

Forests are essential for global climate regulation [1]; however, significant uncertainty persists regarding the response of forest-dominated terrestrial carbon sinks to climate change [2]. The physiological reactions of trees to drought conditions are key factors contributing to this uncertainty [3]. In numerous forested areas worldwide, the likelihood of severe drought is increasing [4, 5], often occurring alongside increased precipitation levels [6, 7]. Droughts exacerbated by climate change have impacted forests globally and are expected to remain a significant factor influencing forest dynamics [8, 9]. Critical aspects of this phenomenon include the capacity of a tree to sustain growth during drought (resistance), enhance growth in relation to the drought’s lowest point (recovery), and return to its pre-drought growth rate (resilience) [10]. Plants exhibit varying degrees of drought tolerance, and strategies have been developed to enhance their ability to withstand water scarcity. Severe drought can lead to dehydration, causing wilting and, ultimately, plant death [11]. Water scarcity, primarily caused by drought, profoundly affects plant growth, development, reproduction, and survival. Understanding plant responses to drought stress is crucial for addressing the water shortage issues related to desertification. The perception of water scarcity begins at the molecular and cellular levels in plant roots, with signals transmitted to the shoots to prepare for challenging conditions. This response involves stomatal closure, energy depletion, and deterioration of plant growth [12]. Stress conditions elicit coordinated responses in plants, influence gene expression, and modify proteins and metabolites [13–15]. Therefore, researchers have aimed to select and develop drought-tolerant plants to address the challenges posed by drought.

Numerous studies have explored the effects of drought stress on plants and their adaptive mechanisms. These studies identified changes in crop yield, growth, pigment synthesis, photosynthetic activity, membrane integrity, osmotic adjustment, stomatal regulation, cell division, and reactive oxygen species (ROS) accumulation in response to drought [16–18]. Drought stress compromises water-use efficiency and hinders CO_2_ assimilation by leaves owing to factors such as stomatal closure, membrane damage, and altered enzyme function [19]. Increased flux through the photorespiratory pathway during stress generates ROS, leading to oxidative damage to nucleic acids, proteins, and lipids [20].

The seedling stage is crucial for plants, influencing their survival and growth throughout their lifespan [21, 22]. Given the prolonged growth period of trees, selecting drought-tolerant varieties at the seedling stage is vital for cost and time efficiency. Researchers have focused on studying the physiological and metabolic responses of seedlings to drought stress to identify screening factors for drought resistance. Improving the survival rate of seedlings is essential for successful afforestation. Therefore, a deeper understanding of drought response during the seedling stage is essential.

*Fraxinus*, commonly known as ash trees, comprises 48 accepted tree and shrubby species distributed across the northern hemisphere [23]. Belonging to the Oleaceae family, *Fraxinus* has been utilized globally in folk medicine for its medicinal properties [24]. This genus, characterized by large imparipinnate leaves and one-seeded samaras, includes four species and one variety from Korea (*Fraxinus chiisanensis* Nakai [FC], *F. mandshurica* Rupr. [FM], *F. rhynchophylla* Hance [FR], *F. sieboldiana* Blume [FS], and *F. sieboldiana var. angustata* Blume [FSV]) [24]. Notably, *F. chiisanensis* Nakai, an endangered and endemic species in southwestern Korea, exhibits unique features such as a naked terminal bud and wind-pollinated flowers [23]. Conservation genetic studies have highlighted the endangered status of *F. chiisanensis*, emphasizing its endemic nature in South Korea [23]. *F. rhynchophylla*, commonly known as East Asian ash, thrives in the moist and fertile soils of hillsides and river valleys across Korea, China, and Japan [25]. It is widely distributed in Korea and has exhibited robust growth, particularly in the mid- to northern regions of the peninsula [26]. This deciduous species, reaching up to 30 m in height and 50 cm in diameter, favors fertile sandy loam soils with adequate moisture and shows resilience to cold temperatures. Serving a crucial ecological role, *F. rhynchophylla* naturally regenerates and dominates after thinning, aiding in the restoration of native hardwood forests [25]. Beyond its ecological contributions, the species provides wildlife habitat, stabilizes stream banks, and contributes organic matter to the forests. Additionally, its bark has medicinal value, whereas its wood is employed in manufacturing furniture and tools owing to its desirable properties [25, 27–29]. However, scientifically validated tolerance rankings for many genotypes within the genus are currently lacking. Previous studies on drought tolerance have identified *F. excelsior* ‘*Aurea Pendula*,’ *F. nigra Marsh.*, *F. ornus L.*, *F. angustifolia* ‘*Raywood*,’ *F. excelsior L.*, and *F. excelsior* ‘*Jaspidea*’ as suitable candidates for urban planting in areas with limited water availability; other *Fraxinus* species have shown a marked sensitivity to drought conditions [30]. Transcriptome analysis of salt tolerance-related genes in *F. velutina* showed the upregulation of starch- and sucrose-related genes in roots, genes related to hormone and MAPK signaling in leaves, and peroxidase (POD)-related genes in both tissues [31]. Additionally, researchers recently screened green ash (*F. pennsylvanica*) for differentially expressed genes (DEGs) by treating it with environmental stress and reported sequence information, genetic markers, and stress response candidate genes [32]. Ash exhibits a tolerance strategy in response to moisture stress, with growth notably affected in areas where water is limited. Previous studies have highlighted a close correlation between growth and foliar nitrogen levels in ash trees under such conditions [33]. *F. mandshurica* demonstrates anisohydric behavior with a negative stomatal safety margin. It exhibits robust resistance to embolisms in both stem and leaf-stem segmentation, emphasizing its capacity to preserve hydraulic integrity under challenging conditions [34].

Among these ash species, we selected *F. chiisanensis*, which is distributed in relatively dry areas, and *F. rhynchophylla*, which is distributed in relatively humid areas. The experiments were planned with the expectation that differences in the distribution of these two species would result in differences in drought tolerance and recovery within ash trees. Specifically, this study investigated the response of ash seedlings to extended drought stress and their recovery after re-watering. We examined the phenotypic and physiological changes in both plants and analyzed the content of typical drought stress indicators and antioxidants. Transcriptome analysis of genes related to drought stress and recovery was also conducted. This study is the first to provide genetic and physiological evidence of the negative impact of medium-term drought on two ash species and their recovery upon rehydration. Ultimately, this study aimed to comprehensively understand how ash trees respond phenotypically, genetically, and physiologically to drought stress and how they exhibit some level of recovery upon rehydration.

## Materials and methods

### Plant materials and growth conditions

The experiment was conducted over 2 months at the National Institute of Forest Science in Suwon (37_1500400 N, 136_5705900 E), Korea, under semicontrolled conditions. Ash seeds were collected from a seed orchard managed by the Forest Bioresources Department of NIFoS, located in Hwaseong-si, Gyeonggi Province, Korea (37.271316 N, 126.922605E) and the Gene Bank of the National Institute of Forest Science in Suwon -si, Gyeonggi Province, Korea (37.251930 N, 126.959548E). Ash seedlings were initially sown in the open field in 2020, replanted in 2021, and then transplanted into pots for the experiment in 2022. The experiment commenced after a two-month acclimatization period. The seedlings were grown in pots (perlite and vermiculite [2:1:1], 11 cm × 11 cm × 3.14 × 24 cm) with a top-soil and sand mixture (3:1), and the greenhouse temperature was maintained between 22 and 29 ℃. Each treatment group, control (watered every two or three days) and drought (no water for 31–36 days), consisted of three replicates (control: 3 replicates × 10 plants; drought: 3 replicates × 10 plants), with 10 plants pooled for analysis in each replicate. Soil moisture levels were monitored regularly using a moisture probe (ICT International Pty. Ltd., Armidale, NSW, Australia). The drought treatment involved withholding water for 31–36 days, and when the soil moisture content dropped to less than 3%, it was maintained at approximately 20–30% after re-watering for 6 days. Ash leaves were harvested between May and July 2022. The upper and lower leaves were harvested and mixed at the same stage. All samples used in the experiments were stored at ‒80 ℃ in a cryotic refrigerator until further analysis.

### Thermal imaging and measurement of the relative water content (RWC) in leaves

Leaf temperature was measured by acquiring infrared digital images using a Fluke TiX560 thermal imaging camera (Fluke Corp., Everett, WA, USA) in the control and drought treatment groups on days 1 and 6 of water resupply.

The RWC (%) was calculated as the ratio of the water content in a leaf’s normal growth state to its maximum water content. The fresh weight (FW) of the plant leaves was measured after immersion in distilled water for 24 h. After removing the surface water, the saturated weight (TW) was measured. Upon drying in a 70 °C dryer for 24 h, the dry weight (DW) of the leaves is determined. The RWC was then calculated using the following formula:

RWC (%) = (FW–DW)/(TW–DW)×100.

where FW is the fresh weight, DW is the dry weight, and TW is the saturated leaf weight.

### Measurement of the chlorophyll content

The efficiency of photosystem II (PSII) was evaluated based on the Fv/Fm ratio and PSII potential activity using the Kautsky induction method [35]. Measurements were performed every 2–6 d on 10 plants at similar growth stages. To measure chlorophyll fluorescence, leaves were dark-adapted for 20 min, followed by irradiation with 1,500 µmol·m⁻²·s⁻¹ for 5 s. Chlorophyll fluorescence variables (Fo, Fm, Fv/Fo, and Fv/Fm) were recorded and analyzed using a Handy Fluorcam instrument (Photon System Instruments Ltd., Brno, Czech Republic) [35, 36].

Photosynthetic pigments were determined following the method described by Sibley et al. (1996) [37]. Briefly, 0.1 g samples of fresh leaves were collected in triplicate. Supernatants were measured spectrophotometrically at 470, 647, and 664 nm using a BioSpectrometer (Eppendorf, Hamburg, Germany). The chlorophyll a, b, and carotenoid concentrations were calculated respectively based on the absorbance values and reported in mg/g fresh weight (FW). The formulas used are:

- Chlorophyll a (Chl a) = 12.7A_664_–2.79A_647_.
- Chlorophyll b (Chl b) = 20.7A_647_–4.62A_664_.
- Carotenoids = (1000A_470_–1.82Chl a–85.02Chl b)/198.

### Extraction and measurement of soluble sugar

For total soluble sugar extraction, 0.1 g of fresh leaves were collected in triplicates and treated with 80% ethanol using the modified Irigoyen method [38]. A BioSpectrometer (Eppendorf) was used to measure the total soluble sugar content at 620 nm, with glucose as the standard, and the results were expressed as mg/g FW. Glucose, fructose, and sucrose were extracted from 0.1 g of fresh leaves in triplicate using the method described by Lu and Sharkey [39]. Concentrations were measured using a BioSpectrometer (Eppendorf), according to Stitt et al. [40]. Starch content analysis involved autoclaving sediments from aqueous ethanol extractions, followed by enzymatic digestion to glucose using α-amylase and amyloglucosidase from the Total Starch Kit (Megazyme International Ireland Ltd., Wicklow, Ireland, K-TSTA-100 A). Sugar concentrations were enzymatically determined using a BioSpectrometer (Eppendorf) following the method of Walters et al. [41] and the approach of Stitt et al. [40].

### Measurement of Malondialdehyde (MDA), Proline, Hydrogen Peroxide (H_2_O_2_), Superoxide Dismutase (SOD), Catalase (CAT), Ascorbate Peroxidase (APX), Peroxidase (POD), Abscisic Acid (ABA), and Indole-3-acetic Acid (IAA) Levels

For proline extraction, 0.5 g of fresh leaves underwent homogenization in a 3% (w/v) aqueous sulfosalicylic acid solution. The proline content was assessed using the ninhydrin reagent method described by Bates et al. [42]. The absorbance was measured at 520 nm using a BioSpectrometer (Eppendorf). Proline concentrations were determined using a calibration curve (expressed as mmol proline/g FW). Malondialdehyde (MDA) measurements involved leaf extraction with a mixture of 20% TCA (w/v) and 0.5% thiobarbituric acid (TBA) (w/v). After heating and centrifugation, the absorbance of the supernatant was measured at 532 nm using a BioSpectrometer (Eppendorf). MDA content was determined using the method described by Heath and Packer [43]. Hydrogen peroxide (H_2_O_2_) was quantified by reacting with 1 M KI and 100 mM K-phosphate buffer (pH 7.0). The absorbance was measured at 390 nm using a BioSpectrometer (Eppendorf), and the amount of H_2_O_2_ was calculated using a standard curve [44]. Enzymatic activity was determined using specific assay kits following the respective manufacturer’s instructions. Specifically, SOD (cat. DG-SOD400; Dogen, Seoul, Korea) and CAT assay kits (cat. DG-CAT400, Dogen), a plant ascorbate peroxidase (APX) kit (MBS2602897, MyBioSource, San Diego, CA, USA), and a plant peroxidase (POD) ELISA kit (MBS9313803, MyBioSource) were used. Optical density was measured at specific wavelengths using an automated plate reader (SpectraMax M2, Molecular Devices, San Jose, CA, USA). The plant hormones IAA and ABA were quantified using a plant IAA ELISA kit (MBS269958, MyBioSource) and plant ABA ELISA kit (MBS703081, MyBioSource), respectively, following the manufacturer’s instructions. The optical density was measured at specific wavelengths using an automated plate reader (SpectraMax M2; Molecular Devices).

### RNA isolation, library preparation, RNA sequencing, differentially expressed gene (DEG) analysis and qPCR analysis

Total RNA was extracted and sequenced to understand gene regulation during drought stress. For this purpose, three replicates from each species, *F. rhynchophylla* and *F. chiianensis*, were used to separate total RNA from the control, drought, and recovered (rewatered) plants at two different timelines: 1 d and 6 d. Following the instructions provided by the manufacturer, either an RNeasy Plant Mini Kit from Qiagen (Hilden, Germany) or a Beniprep^®^ Super Plants RNA Extraction Kit from Invirustech (Gwangju, Korea) was used. A NanoDrop ND–1000 (NanoDrop Technologies, Wilmington, DE, USA) was used to analyze the purity of the isolated RNA. DNA contamination was removed using RNase-free DNase (Promega, Madison, WI, USA). Double-stranded cDNA synthesis was performed using the TaKaRa cDNA EcoDryTM Premix (Shiga, Japan) with equal volumes of high-quality RNA from each sample. The Illumina TruSeq Standard RNA Prep Kit (cat. #RS-122-2103; Illumina, San Diego, CA, USA) was used to prepare cDNA libraries, and 24 samples were sequenced on the Illumina Novaseq 6000 platform from DNAlink, Inc., Seoul, Korea (http://www.dnalink.com/).

Real-time quantitative reverse transcription PCR (qPCR) was performed using a CFX96 Touch Real-Time PCR Detection System (Bio-Rad, Hercules, CA, USA) and an IQTM SYBR Green Supermix (Bio-Rad). The qPCR reaction conditions followed the manufacturer’s instructions, with an initial denaturation at 95 °C for 30 s, followed by 38 cycles of denaturation at 95 °C for 5 s and annealing/extension at 60 °C for 34 s. Three independent biological replicates, each containing three technical replicates, were used. The relative transcript abundance was analyzed using the 2^−ΔΔCt^ method [45]. Actin and 18 S rRNA were used as internal reference genes for normalization (Supplementary Table 1) [31, 46, 47].

FastQC (version 0.11.2) was used to process the quality of the raw RNA-seq data [48]. Trimmomatic (0.40) was used to eliminate low-quality reads and their adapters to obtain clean read data [49]. Using default parameters, the Trinity software (version 2.14.0) was used to generate high-quality reads for *de novo* transcriptome assembly [50]. In summary, Trinity was used to combine the specific lengths of overlapping reads with paired-end data to create longer segments, generating transcripts and unigenes that were then analyzed for annotations. The completeness of the assembled transcriptome was assessed using Benchmarking Universal Single-Copy Orthologs (BUSCO) (v5) on the gVolante web server [51]. Transdecoder (version 2.0.1) (https://github.com/TransDecoder/TransDecoder), accessed on 01 August 2024, was used to convert the contigs into coding protein sequences once the longest open reading frames were identified. Principal component analysis (PCA) based on Spearman’s correlation coefficient was performed as a correlation index between biological replicates by pairwise comparison of samples, and the PCA plot was generated using pcaExplorer (http://shiny.imbei.uni-mainz.de:3838/pcaExplorer/) by loading the count data matrix along with its metadata information [52]. Using CD-HIT version 1.2, we eliminated redundant sequences, found non-redundant or representative protein sequences, and retained the longest sequence in each cluster with a minimum sequence identity threshold of 0.9 contigs [53]. Non-redundant or representative sequences were annotated based on sequence similarity. Supplementary Table 2 provides an overview of the RNA-seq statistics for *F. rhynchophylla* and *F. chiisanensis*. The raw data were deposited in the NCBI Short Read Archive database under the accession number PRJNA1172350.

### Analysis and functional annotation of the DEGs

The transcriptomes of both *Fraxinus* species were mapped back against their raw reads to assess assembly quality; the resulting mapping percentage or alignment percentage is shown in Supplementary Fig. 1. Four comparisons were considered in this study for both *Fraxinus* species: control vs. drought (C vs. D), drought vs. recovery at day 1 (D vs. R1), drought vs. recovery at day 6 (D vs. R6), and control vs. recovery at day 6 (C vs. R6). With an adjusted p-value < 0.05, upregulated and downregulated DEGs were identified based on log_2_ FC ≥ 2 and log_2_ FC ≤ -2, respectively. The assemblies were aligned with the National Center for Biotechnology Information (NCBI) non-redundant protein sequence (TAIR10.1) of *Arabidopsis thaliana* using OmicsBox BLASTX with an evaluation cut-off of 1e-5 against the search database. The Gene Ontology (GO) database OmicsBox (version 3.1) was used for GO mapping. Furthermore, potential signaling pathways in the transcriptomes of *F. rhynchophylla* and *F. chiisanensis* were identified via Kyoto Encyclopedia of Genes and Genomes (KEGG) analysis. Using OmicBox, GO keywords were assigned to the annotated sequences to predict the functions of the various sequences and the encoded translated proteins. A *P-*value ≤ 0.05 was set as the threshold for significance for both the KEGG pathways and the GO keywords when examining the DEGs for functional GO categorization and enrichment analysis [54].

### Construction of Co-expression networks

The WGCNA (version 1.1.75-2) package in R was used to construct co-expression networks, as described by Langfelder and Horvath [55]. This tool facilitates the import of gene expression data and utilizes an automated network construction function, blockwise, to generate co-expression modules. The modules were created using default parameters, except for a minimum module size of 50, a power value of 14, and an unsigned TOMType. Hub transcription factors (TFs) were selected from the significant modules by calculating the correlation coefficients between module eigengenes and physiological data, as outlined by Huang et al. [56]. The networks were visualized using Cytoscape version 3.9.1, following the methodology described by Shannon et al. [57].

### Statistical analysis

Analyses were conducted using a one-way ANOVA with multiple comparisons using Tukey’s honest significant difference test. *P*-values < 0.05 were considered significant. Data are presented as the means and standard deviations (SDs).

## Results

### Growth and physiological changes in response to drought and Re-watering

**Fig. 1:**
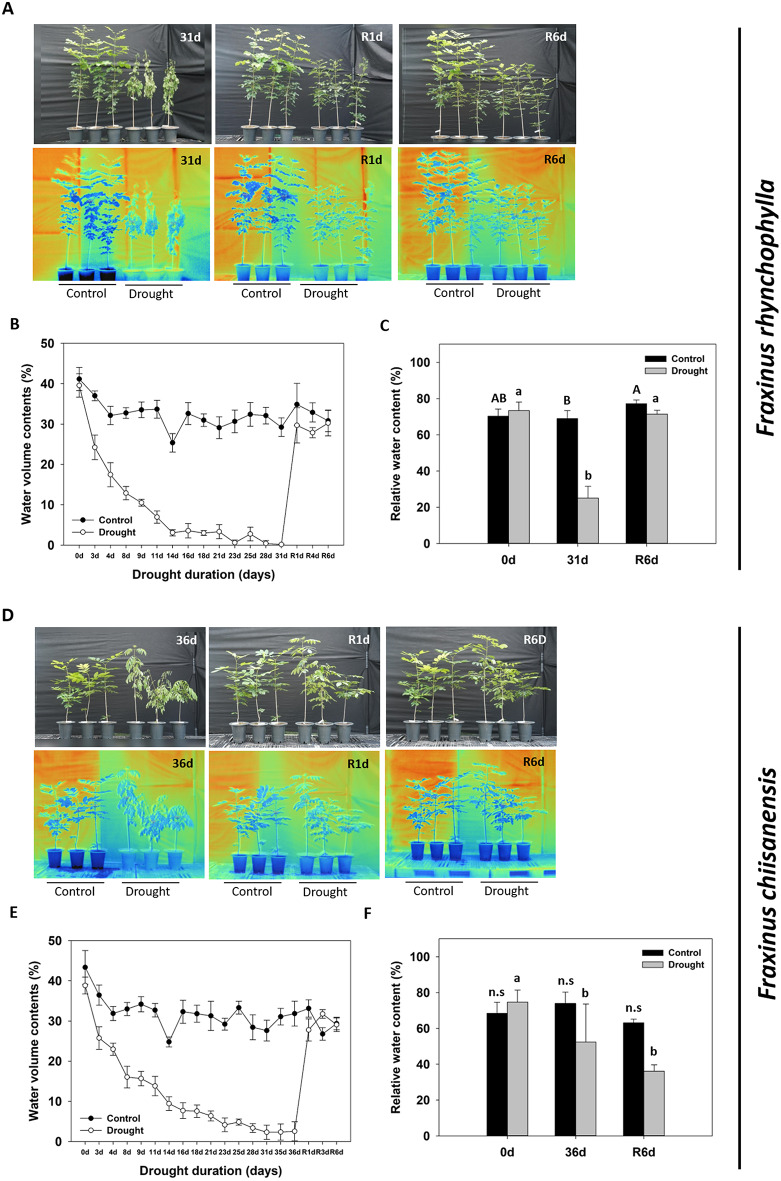
Representative phenotypes of *F. rhynchophylla* and *F. chiisanensis*. (**A**, **D**) Recovery of drought-stressed and control group seedlings after water resupply (upper) and infrared thermal images (bottom). (**B**, **E**) Volumetric water content of the soil in pots containing drought-treated plants. (**C**, **F**) Relative water content (RWC) of the leaves. The values are the means ± SD (*n* = 10). Different uppercase letters and lowercase letters indicate significant differences (control: uppercase letters; drought: lowercase letters; ANOVA with Tukey’s honestly significant difference test, *p* < 0.05)

**Fig. 2:**
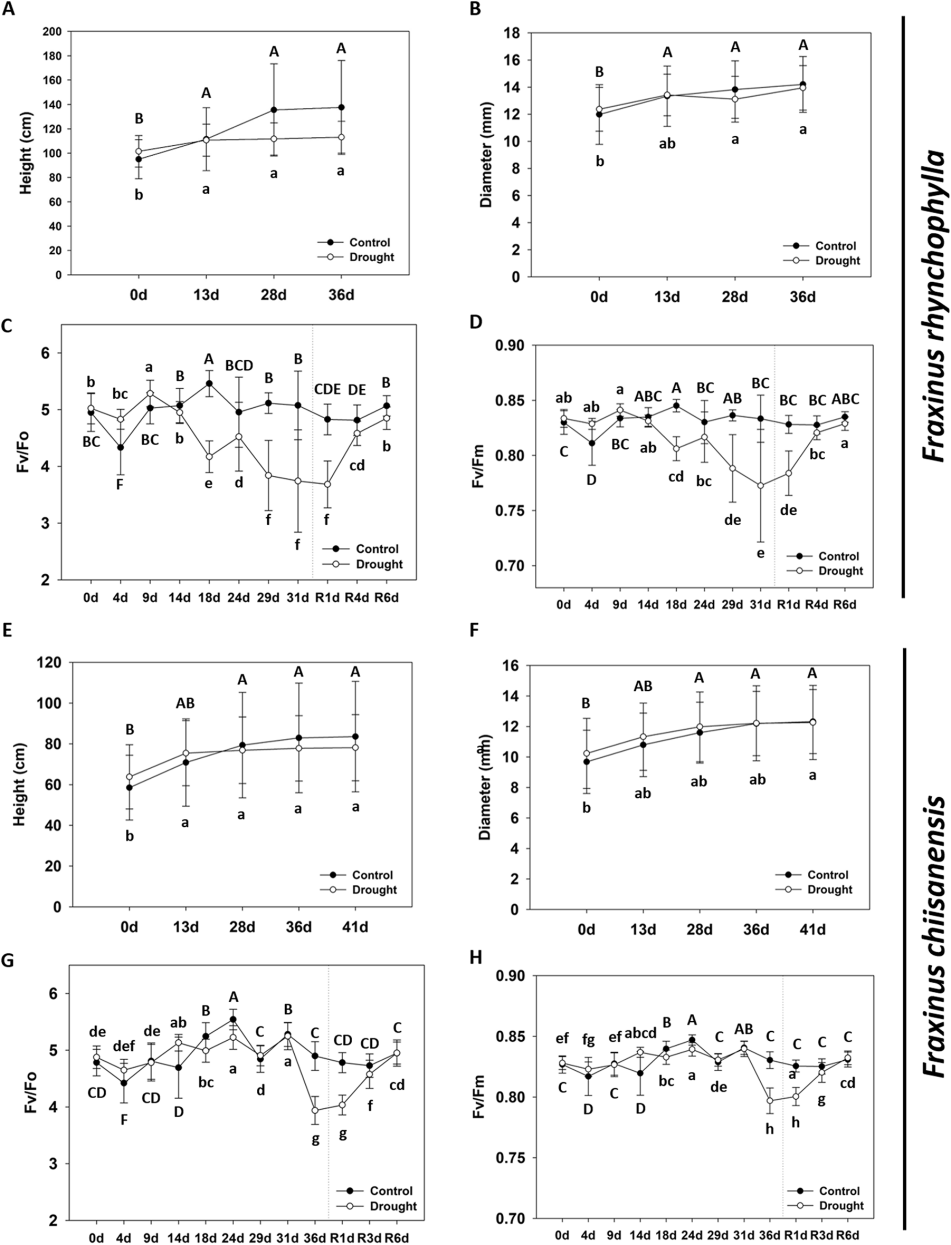
Growth phenotype and physiological changes in *F. rhynchophylla* and *F. chiisanensis*. (**A**,** E**) Effect of drought treatment and re-watering on shoot growth. (**B**,** F**) Effect of drought treatment and re-watering on plant diameter. (**C**,** G**) The Fv/Fo ratio. (**D**,** H**) The Fv/Fm ratio. The values are the means ± SD (*n* = 10). Different uppercase and lowercase letters indicate significant differences (control: uppercase letters; drought: lowercase letters; ANOVA with Tukey’s honestly significant difference test, *p* < 0.05)

When comparing well-watered control and drought-stressed seedlings of *F. rhynchophylla* using a thermal imaging camera, the drought-stressed group exhibited a temperature increase of approximately 1.22 °C (Fig. 1A and B). After water resupply, the temperature difference decreased to approximately 0.4° C on the 6th day. For *F. chiisanensis*, comparing the two treatment groups revealed that the temperature of the drought treatment group was approximately 0.34 °C higher than that of the control (Fig. 1D and E). On the 6th day after water resupply, the temperature of the drought treatment group decreased further to -0.64 °C, indicating a lower temperature than the control. In the RWC analysis of *F. rhynchophylla* leaves, those subjected to drought stress significantly decreased from approximately 70% to approximately 25% (Fig. 1C). However, they recovered to their original levels after water was resupplied. In contrast, the RWC of *F. chiisanensis* leaves decreased from approximately 73–52% under drought stress; even after water resupply, they further decreased to approximately 36% without recovery (Fig. 1F). When comparing the height of *F. rhynchophylla* on the 31st day, the height of the drought treatment group was measured to be approximately 17.98% smaller than the control, confirming a substantial reduction in growth (Fig. 2A). However, no significant difference was observed in the diameter (Fig. 2B). For *F. chiisanensis* on day 36, comparison the two treatment groups revealed that the height of the drought treatment group was approximately 5.17% smaller, with no significant difference in diameter (Fig. 2E and F). Analysis of the chlorophyll fluorescence of *F. rhynchophylla* and examination of changes in the Fv/Fo and Fv/Fm showed that the initial Fv/Fm was approximately 0.83 and significantly decreased from the 18th day, reaching approximately 0.77 on the 31st day (Fig. 2C and D). After water resupply, it gradually recovered, almost returning to normal levels on the 6th day. The Fv/Fo exhibited a similar decline and recovery. *F. chiisanensis* required a longer period for the Fv/Fo and Fv/Fm to decline (Fig. 2G and H). Fv/Fm started at about 0.83 and decreased to approximately 0.79 on the 36th day of drought stress treatment, recovering to almost the same level as the control group on the 6th day after water resupply. Fv/Fo also showed a decline and recovery in a similar manner.

### Drought stress indicators and metabolites change in response to drought and Re-watering

Changes in chlorophyll content in response to drought stress were measured (Table 1). On the last day of drought treatment, chlorophyll a, chlorophyll b, total chlorophyll, and carotenoids in the drought treatment group increased by approximately 48.1%, 66.7%, 56.2%, and 52.5%, respectively, in *F. rhynchophylla* compared to the control. Only the levels of chlorophyll a/b decreased by approximately 12.1%. When water was resupplied, the chlorophyll content tended to decrease slightly; however, it was not statistically significant. Moreover, it did not return to the previous level even on the 6th day, maintaining a higher level than that of the control. The change in chlorophyll content of *F. chiisanensis* also showed a similar trend, with chlorophyll a, b, total chlorophyll, and carotenoid levels increasing by 22.7%, 36.3%, 26.3%, and 23.1%, respectively. Similarly, chlorophyll a/b levels decreased by 10.5%. However, it tended to decrease after re-supplying water, although the resulting difference was not statistically significant. Compared to the control, the chlorophyll content was reduced to almost the same level or even lower in some sections (Table 1).

**Table 1:** Effects of drought stress treatment on photosynthetic pigment content in *Fraxinus rhynchophylla* and *Fraxinus chiisanensis*

	Day	Treatment	mg/g FW ^(w)^				Chl a/b^(w)^	Chl/Car^(w)^
			Chl a	Chl b	Total Chl	Carotenoids		
*Fraxinus rhynchophylla*	1	Control^(z)^	1.85 ± 0.07 ^C^	0.38 ± 0.07 ^B^	2.06 ± 0.10 ^B^	0.51 ± 0.01 ^D^	4.99 ± 0.78 ^N.S^	4.04 ± 0.28 ^N.S^
		Drought^(y)^	1.69 ± 0.11 ^c^	0.41 ± 0.03 ^b^	1.96 ± 0.14 ^b^	0.45 ± 0.04 ^c^	4.11 ± 0.26 ^n.s^	4.35 ± 0.15 ^n.s^
	25	Control^(z)^	2.62 ± 0.12 ^A^	0.56 ± 0.06 ^AB^	2.92 ± 0.15 ^A^	0.70 ± 0.03 ^A^	4.68 ± 0.42 ^N.S^	4.19 ± 0.17 ^N.S^
		Drought^(y)^	3.04 ± 0.49 ^ab^	0.77 ± 0.10 ^a^	3.58 ± 0.55 ^a^	0.82 ± 0.15 ^ab^	3.96 ± 0.34 ^n.s^	4.39 ± 0.19 ^n.s^
	31	Control^(z)^	2.35 ± 0.17 ^AB^	0.54 ± 0.08 ^AB^	2.65 ± 0.24 ^A^	0.61 ± 0.04 ^BC^	4.39 ± 0.30 ^N.S^	4.35 ± 0.12 ^N.S^
		Drought^(y)^	3.48 ± 0.34 ^a^	0.90 ± 0.08 ^a^	4.14 ± 0.39 ^a^	0.93 ± 0.09 ^a^	3.86 ± 0.14 ^n.s^	4.43 ± 0.07 ^n.s^
	R1	Control^(z)^	2.17 ± 0.24 ^BC^	0.52 ± 0.14 ^AB^	2.47 ± 0.34 ^AB^	0.56 ± 0.06 ^CD^	4.34 ± 0.81 ^N.S^	4.45 ± 0.46 ^N.S^
		Drought^(y)^	2.48 ± 0.22 ^bc^	0.70 ± 0.15 ^a^	3.02 ± 0.35 ^ab^	0.65 ± 0.05 ^bc^	3.62 ± 0.48 ^n.s^	4.61 ± 0.41 ^n.s^
	R4	Control^(z)^	2.49 ± 0.11 ^AB^	0.64 ± 0.07 ^A^	2.91 ± 0.13 ^A^	0.64 ± 0.03 ^AB^	3.90 ± 0.43 ^N.S^	4.55 ± 0.23 ^N.S^
		Drought^(y)^	3.03 ± 0.84 ^ab^	0.73 ± 0.19 ^a^	3.51 ± 0.93 ^a^	0.81 ± 0.23 ^ab^	4.18 ± 0.40 ^n.s^	4.35 ± 0.28 ^n.s^
	R6	Control^(z)^	2.45 ± 0.14 ^AB^	0.62 ± 0.09 ^A^	2.85 ± 0.21 ^A^	0.64 ± 0.03 ^AB^	4.03 ± 0.41 ^N.S^	4.47 ± 0.24 ^N.S^
		Drought^(y)^	2.82 ± 0.17 ^ab^	0.78 ± 0.08 ^a^	3.36 ± 0.14 ^a^	0.71 ± 0.06 ^abc^	3.67 ± 0.49 ^n.s^	4.72 ± 0.34 ^n.s^
*Fraxinus chiisanensis*	1	Control^(z)^	1.44 ± 0.23 ^D^	0.25 ± 0.03 ^C^	1.61 ± 0.24 ^C^	0.45 ± 0.07 ^B^	5.85 ± 0.50 ^A^	3.58 ± 0.11 ^C^
		Drought^(y)^	1.38 ± 0.15 ^c^	0.20 ± 0.05 ^c^	1.55 ± 0.20 ^c^	0.47 ± 0.05 ^c^	6.95 ± 1.06 ^a^	3.31 ± 0.14 ^b^
	31	Control^(z)^	2.23 ± 0.17 ^A^	0.50 ± 0.12 ^A^	2.48 ± 0.26 ^A^	0.57 ± 0.02 ^A^	4.63 ± 0.81 ^B^	4.34 ± 0.45 ^A^
		Drought^(y)^	2.17 ± 0.20 ^a^	0.40 ± 0.04 ^ab^	2.37 ± 0.22 ^ab^	0.61 ± 0.06 ^ab^	5.42 ± 0.06 ^b^	3.86 ± 0.07 ^a^
	36	Control^(z)^	1.80 ± 0.11 ^BCD^	0.33 ± 0.05 ^BC^	1.98 ± 0.13 ^BC^	0.52 ± 0.02 ^AB^	5.50 ± 0.61 ^AB^	3.80 ± 0.19 ^BC^
		Drought^(y)^	2.21 ± 0.19 ^a^	0.45 ± 0.06 ^a^	2.50 ± 0.23 ^a^	0.64 ± 0.06 ^a^	4.92 ± 0.51 ^b^	3.93 ± 0.11 ^a^
	R1	Control^(z)^	1.86 ± 0.09 ^ABC^	0.36 ± 0.03 ^ABC^	2.03 ± 0.11 ^ABC^	0.51 ± 0.02 ^AB^	5.20 ± 0.27 ^AB^	3.99 ± 0.09 ^ABC^
		Drought^(y)^	1.75 ± 0.28 ^bc^	0.35 ± 0.05 ^b^	2.00 ± 0.30 ^b^	0.52 ± 0.07 ^bc^	4.99 ± 0.14 ^b^	3.84 ± 0.05 ^a^
	R3	Control^(z)^	2.09 ± 0.17 ^AB^	0.44 ± 0.05 ^AB^	2.30 ± 0.19 ^AB^	0.55 ± 0.05 ^AB^	4.71 ± 0.42 ^AB^	4.23 ± 0.14 ^AB^
		Drought^(y)^	1.87 ± 0.06 ^ab^	0.35 ± 0.03 ^b^	2.02 ± 0.06 ^b^	0.51 ± 0.01 ^bc^	5.34 ± 0.32 ^b^	3.95 ± 0.16 ^a^
	R6	Control^(z)^	1.67 ± 0.26 ^CD^	0.32 ± 0.04 ^BC^	1.85 ± 0.28 ^BC^	0.48 ± 0.08 ^AB^	5.20 ± 0.21 ^AB^	3.90 ± 0.09 ^ABC^
		Drought^(y)^	1.87 ± 0.03 ^ab^	0.38 ± 0.02 ^ab^	2.09 ± 0.02 ^ab^	0.53 ± 0.02 ^abc^	4.94 ± 0.33 ^b^	3.98 ± 0.13 ^a^

^(z)^ Control; ^(y)^ Drought; ^(w)^ Values are the means ± SDs (*n* = 3). Different lowercase letters indicate significant differences (ANOVA with Tukey’s honest significant difference, *p* < 0.05)

We also examined how soluble sugars and starch were regulated in response to drought stress in *F. rhynchophylla* and *F. chiisanensis* (Table 2). In F. *rhynchophylla*, glucose and sucrose levels significantly increased by approximately 2.34- and 5-fold, respectively, in the drought-treated group compared to the control, while starch levels decreased by almost half. After water resupply, glucose and sucrose levels gradually decreased, but only by approximately 18.9% and about 39.8%, respectively, compared to the high point, but were still higher than the control. In contrast, fructose levels showed no difference from the previous concentration during drought stress. However, it increased significantly by approximately 4.5 times when water was resupplied and then gradually recovered to the previous level. Starch levels rapidly decreased upon subjection to drought stress, down to approximately 47.1%, but recovered to the previous level in just one day after re-watering. The changes in soluble sugar and starch levels in *F. chiisanensis* were dramatic. Specifically, glucose, fructose, and sucrose levels increased by approximately 2.77-, 8.23-, and 1.16-fold, respectively. After water resupply, glucose and fructose levels were almost at their previous levels and recovered to a level similar to that in the control. However, sucrose concentrations gradually increased. Starch levels decreased significantly to less than 4% compared to the control, but recovered to approximately 75% of the control after re-watering (Table 2).

**Table 2:** Effects of drought stress treatment on carbohydrate contents in *Fraxinus rhynchophylla* and *Fraxinus chiisanensis*

	Day	Treatment	mg/g FW ^(w)^					
			Glucose	Fructose	Sucrose	Starch	Total soluble sugar ^(w)^	
*Fraxinus rhynchophylla*	1	Control^(z)^	16.04 ± 0.74 ^CD^	2.16 ± 0.29 ^A^	12.28 ± 0.81 ^B^	0.61 ± 0.19 ^B^	287.97 ± 16.33 ^AB^	
		Drought^(y)^	14.98 ± 0.08 ^d^	1.97 ± 0.46 ^bc^	9.95 ± 0.42 ^d^	0.57 ± 0.19 ^b^	261.41 ± 12.46 ^d^	
	25	Control^(z)^	15.59 ± 1.34 ^D^	0.73 ± 0.90 ^B^	6.87 ± 1.72 ^C^	0.35 ± 0.03 ^C^	238.29 ± 30.52 ^B^	
		Drought^(y)^	38.83 ± 3.28 ^a^	1.96 ± 0.24 ^bc^	19.54 ± 1.20 ^bc^	0.07 ± 0.01 ^c^	253.40 ± 26.60 ^b^	
	31	Control^(z)^	15.98 ± 0.68 ^CD^	0.46 ± 0.16 ^B^	7.46 ± 0.97 ^C^	0.12 ± 0.01 ^D^	314.06 ± 31.48 ^B^	
		Drought^(y)^	37.42 ± 1.34 ^ab^	1.24 ± 0.32 ^c^	37.28 ± 3.04 ^a^	0.07 ± 0.01 ^c^	260.79 ± 6.84 ^a^	
	R1	Control^(z)^	18.40 ± 1.65 ^BC^	0.96 ± 0.19 ^B^	10.60 ± 0.89 ^B^	0.38 ± 0.05 ^C^	280.55 ± 14.69 ^B^	
		Drought^(y)^	34.94 ± 0.79 ^ab^	5.70 ± 0.45 ^a^	18.87 ± 2.22 ^c^	0.35 ± 0.01 ^b^	530.97 ± 31.42 ^bc^	
	R4	Control^(z)^	22.15 ± 1.06 ^A^	1.10 ± 0.12 ^B^	15.05 ± 0.21 ^A^	0.75 ± 0.04 ^AB^	637.61 ± 53.02 ^A^	
		Drought^(y)^	33.58 ± 2.65 ^bc^	2.19 ± 0.20 ^b^	23.60 ± 2.01 ^b^	1.66 ± 0.12 ^a^	459.74 ± 47.60 ^c^	
	R6	Control^(z)^	20.41 ± 1.46 ^AB^	0.68 ± 0.16 ^B^	11.64 ± 0.76 ^B^	0.83 ± 0.07 ^A^	445.76 ± 14.63 ^B^	
		Drought^(y)^	30.35 ± 1.74 ^c^	1.26 ± 0.26 ^c^	22.43 ± 0.42 ^bc^	1.50 ± 0.12 ^a^	414.54 ± 7.70 ^c^	
*Fraxinus chiisanensis*	1	Control(z)	17.36 ± 3.02 ^A^	0.60 ± 0.08 ^A^	9.06 ± 0.42 ^A^	0.46 ± 0.05 ^C^	169.11 ± 49.47 ^N.S^	
		Drought(y)	19.12 ± 3.14 ^c^	0.96 ± 0.27 ^c^	8.27 ± 0.40 ^b^	0.45 ± 0.04 ^bc^	159.15 ± 22.52 ^c^	
	31	Control(z)	10.19 ± 0.89 ^B^	0.27 ± 0.10 ^B^	7.59 ± 0.56 ^BC^	0.15 ± 0.02 ^D^	137.15 ± 5.44 ^N.S^	
		Drought(y)	25.66 ± 1.36 ^ab^	1.83 ± 0.18 ^b^	8.79 ± 0.62 ^b^	0.05 ± 0.02 ^d^	157.75 ± 14.93 ^a^	
	36	Control(z)	9.57 ± 0.79 ^B^	0.30 ± 0.10 ^B^	7.34 ± 1.00 ^C^	0.79 ± 0.02 ^B^	173.93 ± 48.83 ^N.S^	
		Drought(y)	26.48 ± 0.31 ^a^	2.47 ± 0.25 ^a^	8.51 ± 0.40 ^b^	0.03 ± 0.01 ^d^	146.85 ± 21.30 ^a^	
	R1	Control(z)	11.98 ± 0.61 ^B^	0.36 ± 0.18 ^AB^	9.56 ± 0.45 ^A^	1.22 ± 0.06 ^A^	161.44 ± 40.67 ^N.S^	
		Drought(y)	22.71 ± 1.04 ^b^	2.27 ± 0.35 ^ab^	11.74 ± 0.70 ^a^	0.29 ± 0.05 ^c^	308.13 ± 8.23 ^a^	
	R3	Control(z)	10.01 ± 0.41 ^B^	0.36 ± 0.18 ^AB^	8.93 ± 0.59 ^AB^	0.70 ± 0.05 ^B^	310.08 ± 20.16 ^N.S^	
		Drought(y)	16.71 ± 0.63 ^cd^	0.91 ± 0.27 ^c^	10.92 ± 1.51 ^a^	0.55 ± 0.06 ^b^	293.77 ± 28.76 ^b^	
	R6	Control(z)	10.87 ± 0.52 ^B^	0.32 ± 0.09 ^AB^	7.59 ± 0.42 ^BC^	1.16 ± 0.12 ^A^	243.15 ± 18.88 ^N.S^	
		Drought(y)	15.32 ± 0.67 ^d^	0.57 ± 0.17 ^c^	11.42 ± 0.68 ^a^	0.88 ± 0.23 ^a^	226.93 ± 10.31 ^b^	

^(z)^ Control; ^(y)^ Drought; ^(w)^ Values are the means ± SDs (*n* = 3). Different uppercase and lowercase letters indicate significant differences (ANOVA with Tukey’s honest significant difference, *p* < 0.05)

**Fig. 3:**
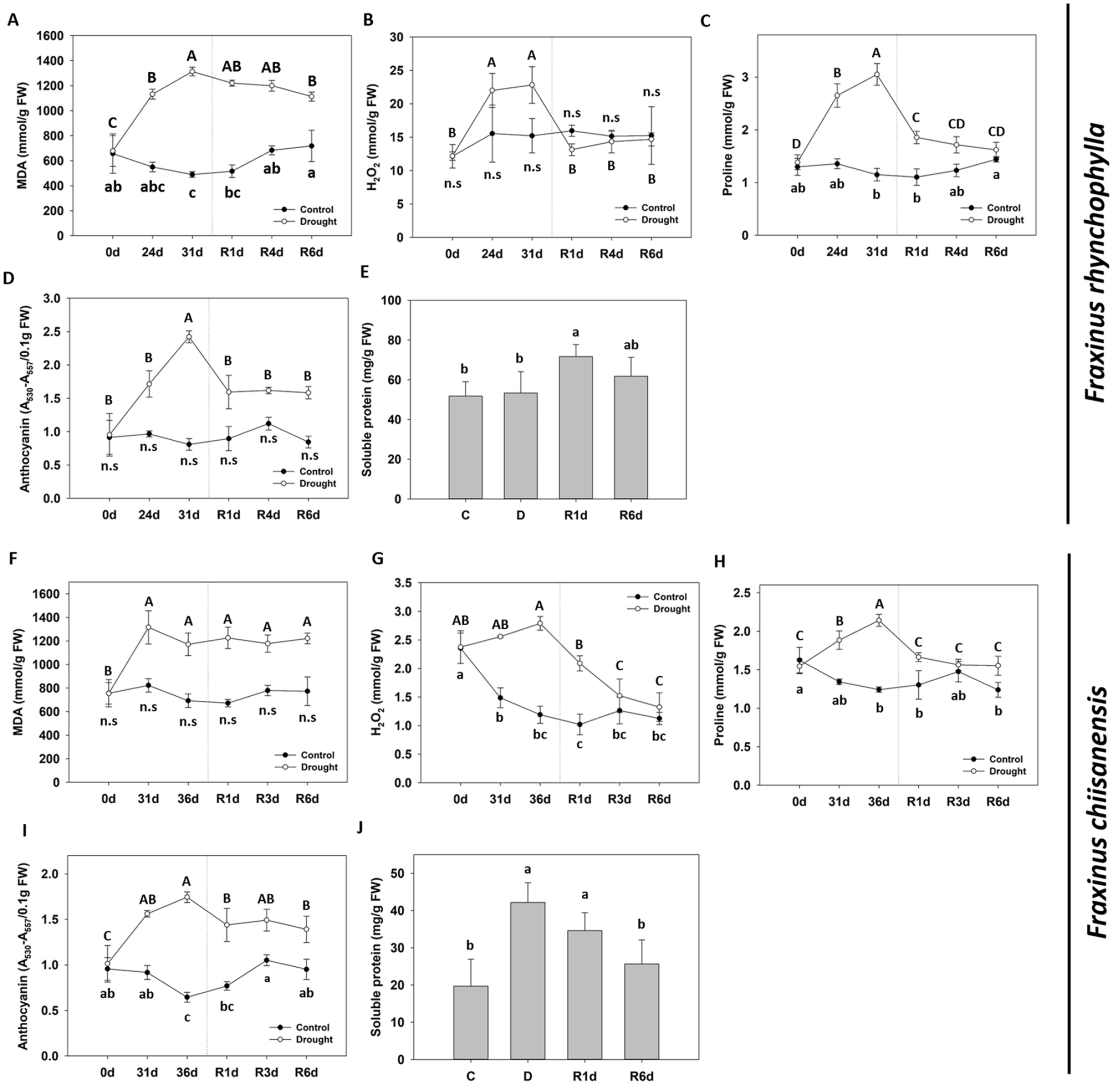
Effect of drought stress and re-watering on drought stress indicators in *F. rhynchophylla* and *F. chiisanensis*. (**A**, **F**) Malondialdehyde (MDA) content. (**B**, **G**) H_2_O_2_ content. (**C**, **H**) Proline content. (**D**, **I**) Anthocyanin content. (**E**, **J**) Soluble protein content. Different uppercase letters and lowercase letters indicate significant differences (control: uppercase letters; drought: lowercase letters; ANOVA with Tukey’s honestly significant difference test, *p* < 0.05)

We measured the changes in the levels of MDA, a representative plant stress indicator (Fig. 3A and F). In *F. rhynchophylla*, MDA levels increased significantly 31 days after drought treatment and only partially decreased after re-watering. MDA levels in *F. chiisanensis* also reached a peak on the 31st day, but did not decrease even after re-watering. We also examined how H_2_O_2_ levels, another representative stress indicator, changed in response to drought stress (Fig. 3B and G). During drought stress, H_2_O_2_ levels in *F. rhynchophylla* gradually increased, fell below that of the control one day after water resupply, and recovered to normal levels by the 6th day. In *F. chiisanensis*, H_2_O_2_ levels rose slightly, but after re-watering, it decreased steeply and recovered to almost the same level as that in the control. The level of proline, a drought stress indicator and osmotic regulator, was also measured (Fig. 3C and H). Proline levels in *F. rhynchophylla* increased very rapidly to approximately 3 times compared to the control on the 31st day. However, they decreased rapidly after water resupply and recovered to a level almost similar to that of the control on the 6th day. In the case of *F. chiisanensis*, proline levels increased by approximately 1.5-fold and then recovered to the previous level one day after water was resupplied. In *F. rhynchophylla*, anthocyanin content increased significantly during drought but was almost normalized when water was resupplied (Fig. 3D). The anthocyanin content in *F. chiisanensis* also increased significantly during drought, decreased slightly after water resupply, and did not completely recover to the previous level (Fig. 3I). *F. rhynchophylla* and *F. chiisanensis* showed different patterns of changes in soluble protein content, which are also indicators of drought stress (Fig. 3E and J). In *F. rhynchophylla*, there was no change in the soluble protein concentration when subjected to drought treatment; however, when water was resupplied, the soluble protein levels increased significantly on the first day and partially decreased on the 6th day. In contrast, in the case of *F. chiisanensis*, the concentration of soluble protein increased significantly (by more than two-fold) under drought conditions and gradually decreased and recovered to the previous level when water was resupplied.

### Changes in hormone and antioxidant levels in response to drought and Re-watering

**Fig. 4:**
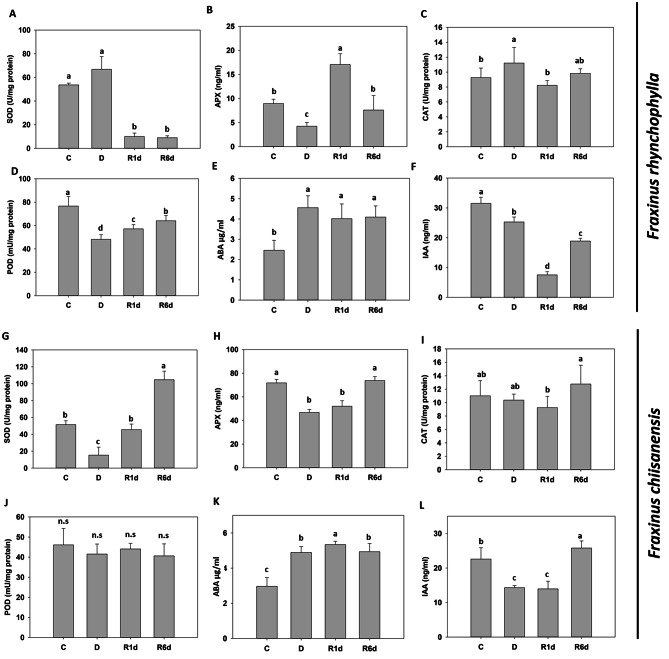
Effect of drought stress and re-watering on drought stress hormones and antioxidants in *F. rhynchophylla* and *F. chiisanensis*. (**A**, **G**) Superoxide dismutase (SOD) activity. (**B**, **H**) Ascorbate peroxidase (APX) activity. (**C**, **I**) Catalase (CAT) activity. (**D**, **J**) Peroxidase (POD) activity. (**E**), (**K**) Abscisic acid (ABA) content. (**F**), (**L**) Indole-3-acetic acid (IAA) content. Different lowercase letters indicate significant differences (ANOVA with Tukey’s honestly significant difference test, *p* < 0.05)

The activities of the antioxidant enzymes SOD, APX, CAT, and POD changed under drought stress and recovery conditions (Fig. 4A, D, G and J). In *F. rhynchophylla*, SOD activity did not change under drought conditions; however, when water was resupplied, its activity decreased sharply and remained in a reduced state, even in R6d. Meanwhile, APX activity decreased under drought conditions, increased significantly just one day after water was resupplied, and recovered to its previously normal level at R6d. CAT activity increased in response to drought and then recovered to control levels starting on R1d. POD activity decreased significantly during drought and slowly recovered after water resupply but did not recover to control levels, even on R6d. In *F. chiisanensis*, SOD activity decreased significantly when subjected to drought but recovered to normal levels on R1d; on R6d, SOD activity significantly increased to almost twice that of the control. APX activity decreased significantly during drought but recovered to the normal level on R6d after water resupply. CAT activity showed no significant difference even under drought conditions; however, after water resupply, it slightly decreased and then increased. POD activity did not show any significant changes across all investigated conditions.

The levels of ABA and IAA, representative hormones involved in the response to drought stress and recovery, were also measured (Fig. 4E, F and K, and 4L). In *F. rhynchophylla*, ABA levels increased significantly under drought conditions and did not decrease until R6d, even after re-watering. IAA levels decreased during drought, decreased more significantly on R1d, and recovered and continued to increase on R6d. At the same time, in *F. chiisanensis*, ABA levels increased significantly during drought, continued to increase for a day even after water resupply, and decreased only slightly on R6d. IAA levels decreased significantly under drought conditions and remained reduced even after water resupply, but recovered by increasing to a greater extent than the previous level in the control on R6d.

### Overview of the transcriptome sequencing and *de Novo* assembly results

A total of ~ 389 million raw reads were generated from the *F. rhynchophylla* leaf transcriptome, while ~ 382 million raw reads were obtained from *F. chiisanensis* using paired-end sequencing, a total of 78.60 GB and 77.17 GB, respectively. The pre-processing of *F. rhynchophylla* raw reads was done to remove adaptor sequences and low-quality reads (Phred score < 30). A total of ~ 372.51 million and ~ 368.3 million clean reads with a Phred score ≥ 30 and GC contents of 41.04% and 44.04% in *F. rhynchophylla* and *F. chiisanensis*, respectively, were retained. The Trinity assembler was employed for the *de novo* assembly of the short-read sequences. In *F. rhynchophylla*, 50,681 assembled transcripts were generated, with a mean size of 10,785 base pairs (bp), whereas in *F. chiisanensis*, 50,674 assembled transcripts were generated, with a mean size of 10,840 bp. The assembled transcripts were further clustered using CD-HIT into 238,870 unigenes in *F. rhynchophylla* and 209,992 unigenes in *F. chiisanensis*. The transcriptome assembly details are listed in Table 3. Among the unigenes, the minimum length was 297 bp in both *Fraxinus* species, while the maximum length was 15,950 bp in *F. rhynchophylla* and 15,276 in *F. chiisanensis* (Table 3).

**Table 3:** Statistics of the transcriptome assembly

Assembly Parameters	F. rhynchophylla	F. chiisanensis
N50	1684	1065
L50	10,785	10,840
Min.	297	297
Max.	15,950	15,276
Average	1163	784
Count	50,681	50,674
Total Assembly length	58,964,113	39,740,628
BUSCO (%)	90.02	92.16
GC %	41.04	44.04

The average GC content of the contigs derived from the transcriptomes of *F. rhynchophylla* and *F. chiisanensis* was 41.04% and 44.04%, respectively (Table 3). The results indicated that the assembly was almost complete, with an adequate representation of the gene directory. The quality of the final assembly was further evaluated through the assessment procedure implemented in BUSCO v. 5.4.4. It provides a quantitative measure of transcriptome quality and completeness based on evolutionarily informed expectations of gene content from near-universal, ultra-conserved protein databases. We analyzed the gene content by launching BUSCO v. 5 in the Eukaryota database (OrthoDB v10). In Table 3, we report transcriptome completeness in BUSCO. Notably, we found a high percentage of complete genes in the eukaryote databases, confirming the good quality of our assembly. In *F. rhynchophylla*, the total core genes queried against the databases were 255, of which only 230 genes (90.20%) were complete, and 243 genes (95.29%) were complete and partial. In *F. chiisanensis*, the total core genes queried against the databases were 255, of which only 235 genes (92.16%) were complete, and 249 genes (97.65%) were complete and partial. Another quality assessment method was implemented by aligning the processed raw data with the final *de novo* transcriptome assembly. The mapping outcomes are shown in Supplementary Fig. 1. The average mapping percentages were 71.61% for *F. rhynchophylla* and 76.34% for *F. chiisanensis*, affirming the high quality of the assembly. Even though mapping rates greater than 90% were considered the best, mapping percentages close to 70% are already acceptable.

### DEGs

Sequencing reads from three replicates of *F. rhynchophylla* and *F. chiisanensis* grown under drought and subsequent recovery conditions revealed significant findings regarding DEGs. In *F. rhynchophylla*, 6,048 DEGs were identified in the comparison of control versus drought stress (C vs. D), 4,473 DEGs in drought versus recovery stage 1 (D vs. R1), 5,365 DEGs in drought versus recovery stage 2 (D vs. R6), and 1,317 DEGs in control versus recovery stage 2 (C vs. R6) (Supplementary Table 3). In *F. chiisanensis*, 3,914 DEGs were found in C vs. D, 3,770 in D vs. R1, 3,288 in D vs. R6, and 821 in C vs. R6. On average, 3,625 DEGs were regulated across both *Fraxinus* species. Specifically, *F. rhynchophylla* exhibited 8,362 upregulated and 8,841 downregulated DEGs, whereas *F. chiisanensis* showed 6,312 upregulated and 5,481 downregulated DEGs. Notably, the overall differential expression rate of the DEGs was lower in *F. chiisanensis* than in *F. rhynchophylla*.

**Fig. 5:**
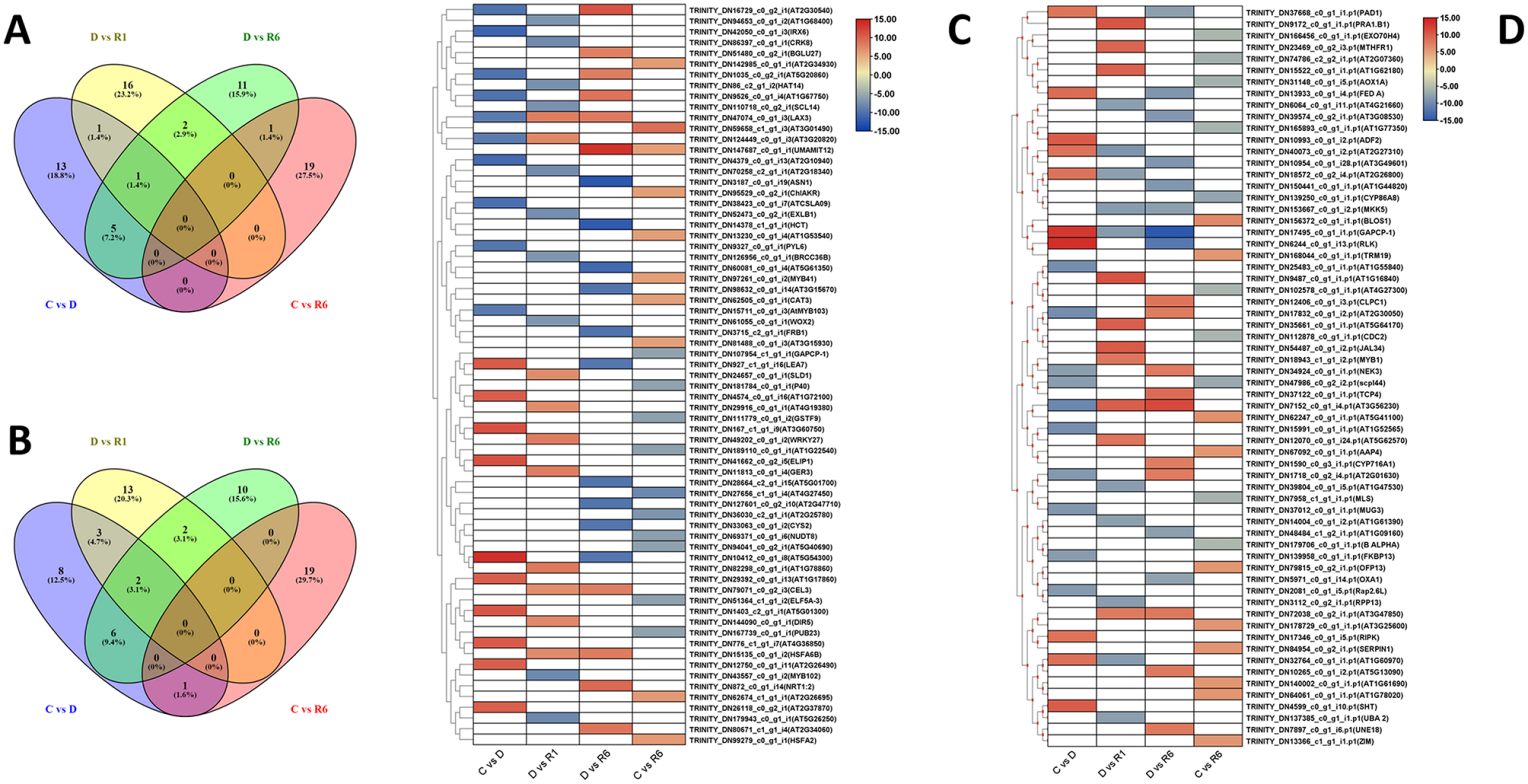
Identification of differentially expressed genes (DEGs) between *F. rhynchophylla* and *F. chiisanensis* induced in response to drought and re-watering. The Venn diagram displays (**A**) the number of DEGs across the comparisons C vs. D, D vs. R1, D vs. R6, and C vs. R6 in *F. rhynchophylla*; (**B**) the number of DEGs across the comparisons C vs. D, D vs. R1, D vs. R6, and C vs. R6 in *F. chiisanensis*. (**C**) Heatmap showing the top 10 DEGs in the comparison groups C vs. D, D vs. R1, D vs. R6, and C vs. R6 in *F. rhynchophylla*. (**D**) Heatmap showing the top 10 DEGs in the comparison groups C vs. D, D vs. R1, D vs. R6, and C vs. R6 in *F. chiisanensis*

This study also examined the differential expression of genes across various metabolic pathways to elucidate their roles under drought and re-watering conditions. The PCA and correlation analyses conducted in this study indicated a strong positive correlation among the sample groups, which were categorized into distinct groups, including *F. rhynchophylla* and *F. chiisanensis*, at both the gene and sample levels (Supplementary Fig. 2A and 3 A). A heatmap illustrating the pairwise correlations among various plant samples is presented in Supplementary Fig. 2B and 3B. A volcano plot of the transcriptome-wide analysis of both *Fraxinus* species is shown in Supplementary Fig. 4. Venn diagrams showing the number of common and specific DEGs are shown in Fig. 5A and B. The ten most significantly upregulated and downregulated DEGs were identified according to the log2 FoldChange values and adjusted *p*-values < 0.05 and are illustrated as a heatmap in Fig. 5C and D.

### Functional annotation of the unigenes

**Fig. 6:**
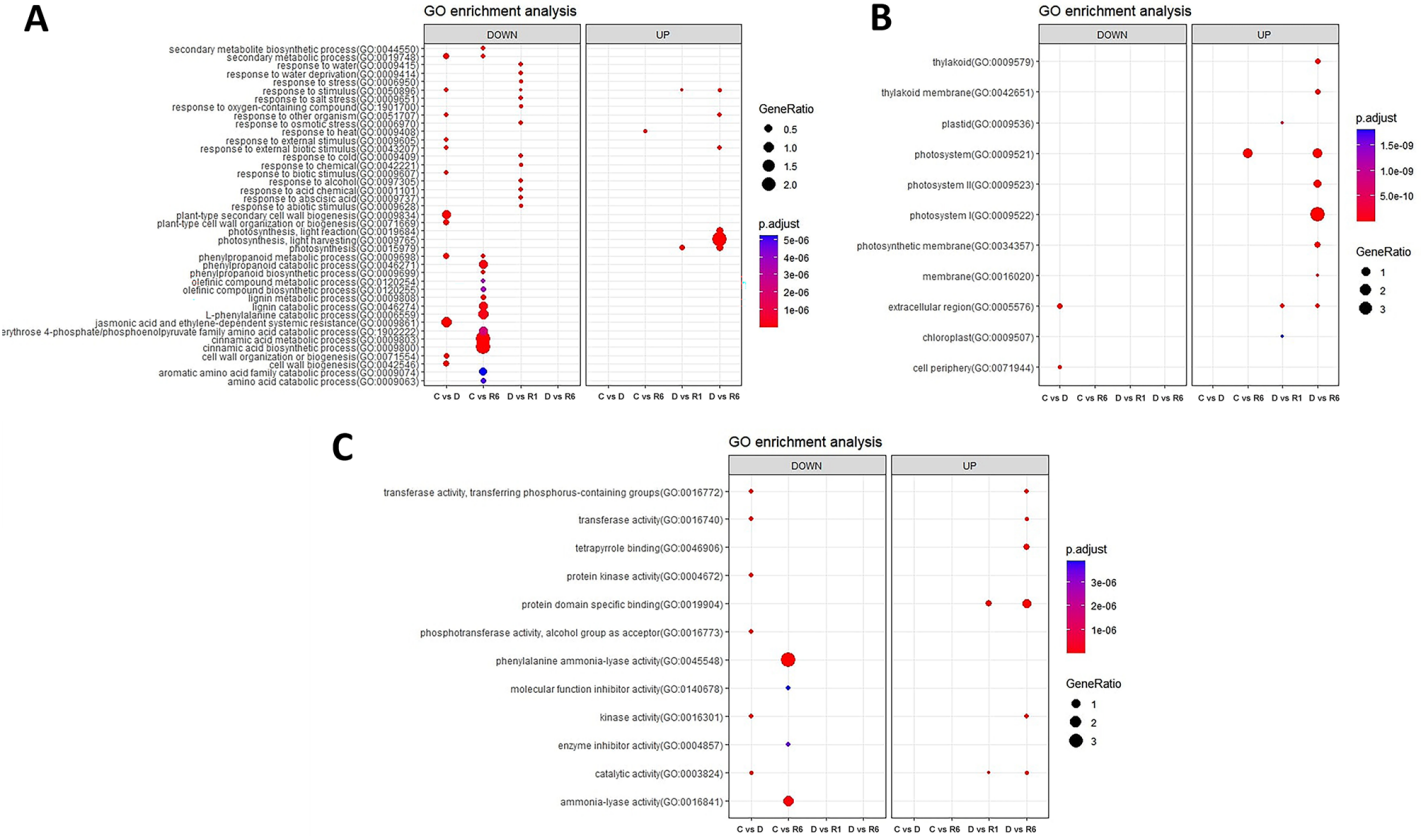
Dot plots illustrating the 10 most significant Gene Ontology (GO) terms identified in the GO enrichment analysis. (**A**) Dot plot representing the top 10 GO terms for the biological process category. (**B**) Dot plot depicting the top 10 GO terms for the cellular component category. (**C**) Dot plot showing the top 10 GO terms in *F. rhynchophylla* for the molecular function category

**Fig. 7:**
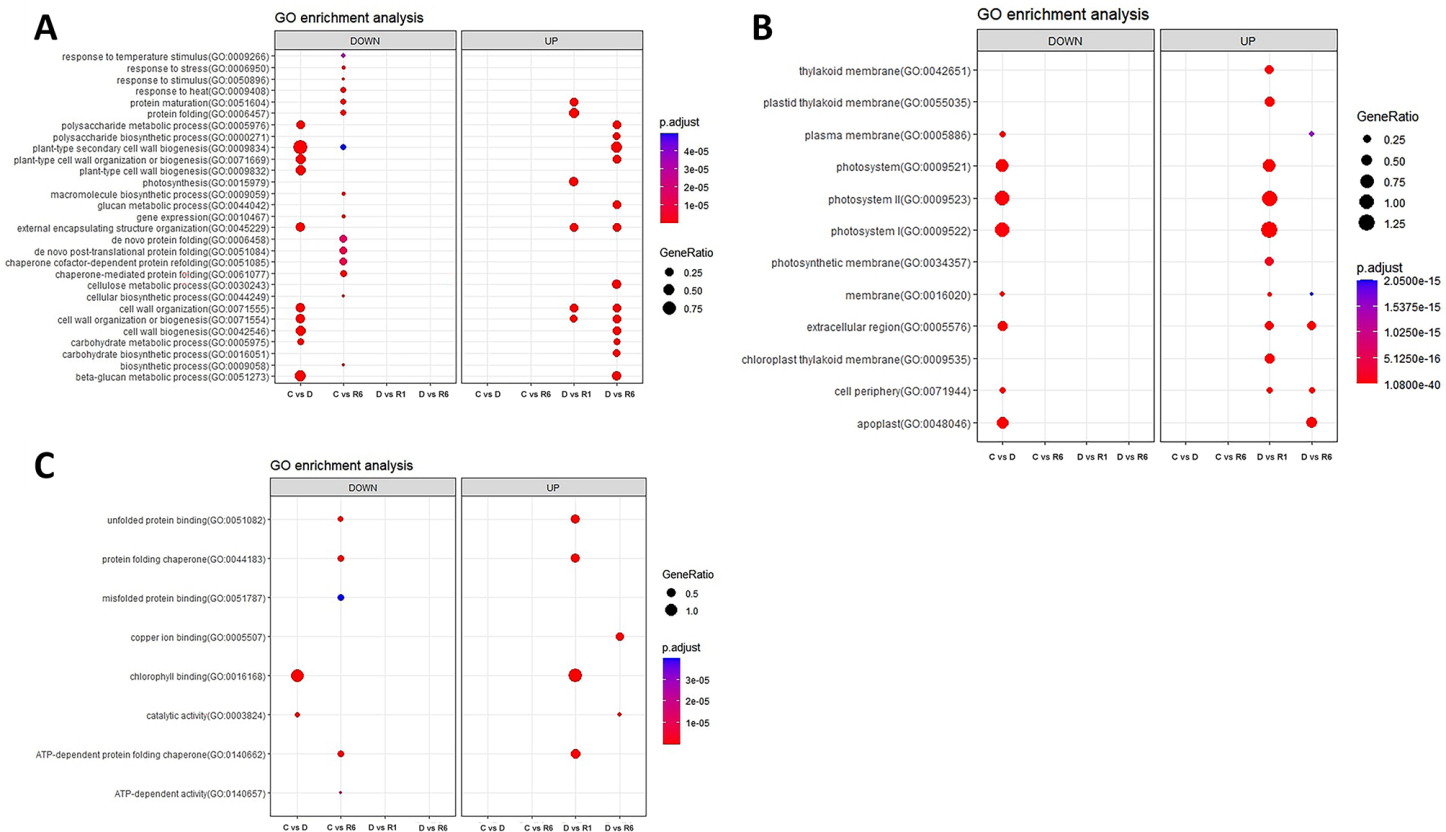
Dot plots illustrating the 10 most significant Gene Ontology (GO) terms identified in the GO enrichment analysis. (**A**) Dot plot representing the top 10 GO terms for the biological process category. (**B**) Dot plot depicting the top 10 GO terms for the cellular component category. (**C**) Dot plot showing the top 10 GO terms in *F. chiisanensis* for the molecular function category

**Fig. 8:**
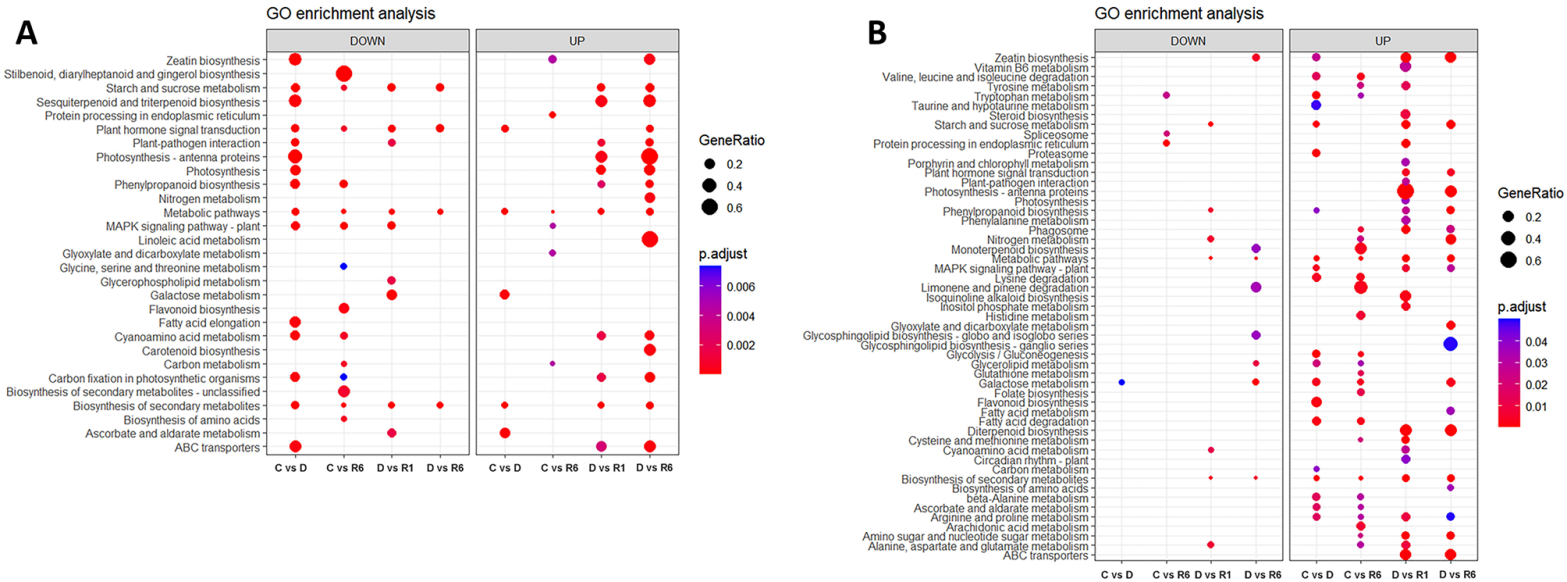
Dot plots illustrating the 10 most significant KEGG terms identified in the enrichment analysis. (**A**) Dot plot representing the top 10 KEGG terms across different comparison groups of *F. rhynchophylla.* (**B**) The dot plot representing the top 10 KEGG terms across different comparison groups of *F. chiisanensis*

The assembled unigenes of *F. rhynchophylla* and *F. chiisanensis* were annotated for sequence similarity search and compared using BLASTx against the protein database of *A. thaliana* (TAIR 10.1) in NCBI with an e-cutoff of 1e-5. The BLASTx results showed the annotation of 34,465 unigenes out of 50,681 assembled transcripts in *F. rhynchophylla* and 33,443 unigenes out of 50,674 assembled transcripts in *F. chiisanensis*. In *F. rhynchophylla*, 66.72% of the unigenes were annotated, 32% were unannotated hits, and approximately 1.3% were uninformative hits (e.g., uncharacterized/hypothetical proteins). In *F. chiisanensis*, 64.73% of the unigenes were annotated, 34% did not show any hits, and 1.26% showed uninformative hits owing to a lack of *Fraxinus* genome information in public databases. Based on BLASTx annotation, the transcripts were subjected to GO and KEGG annotations. OmicsBox is a prominent platform in the field of bioinformatics used for large-scale functional annotation and data mining of sequencing data of non-model species. GO mapping, annotation, and enrichment analyses were performed using OmicsBox. GO terms were collected and processed to construct dot plots and heat maps. Figure 6 shows the GO enrichment data as dot plots across three categories: biological process, cellular component, and molecular function in *F. rhynchophylla.* The data for *F. chiisanensis* are shown in Fig. 7. KEGG enrichment analysis was performed using KOBAS-i (http://kobas.cbi.pku.edu.cn) representation. The dot plot for KEGG enrichment in *F. rhynchophylla* is shown in Fig. 8A, and that for *F. chiisanensis* is shown in Fig. 8B.

### Functional classification of unigenes

In the analysis of GO annotations for *F. rhynchophylla*, 50,681 isoforms were classified into three primary categories: biological processes, cellular components, and molecular functions. Within the biological processes category, the three leading terms based on the false discovery rate (FDR) were “photosynthesis” (GO:0015979), “cell wall organization or biogenesis” (GO:0071554), and “response to stimulus” (GO:0050896). For cellular components, the most represented terms included “photosystem” (GO:0009521), “extracellular region” (GO:000576), and “photosystem I” (GO:000952). In terms of molecular functions, the classifications with the highest number of transcripts were “transferase activity” (GO:0016740), “catalytic activity” (GO:0003824), and “kinase activity” (GO:0016301). In *F. chiisanensis*, 50,674 isoforms were similarly categorized. The top three terms in the biological process category based on the FDR were “stomatal development” (GO:0010118), “response to metal ions” (GO:0010038), and “cellular response to endogenous stimuli” (GO:0071495). For cellular components, the enriched GO terms included “plant-type vacuole” (GO:0000325), “membrane protein complex” (GO:0098796), and “microtubule end” (GO:1990752). The most significantly enriched GO terms associated with DEGs in the comparisons “C vs. D” and “D vs. R6” were linked to enzymatic activity and photosynthesis, respectively (Figs. 6, 7 and 8). KEGG enrichment analysis was also performed to understand the vital pathways regulated during the exposure to drought. The enriched KEGG pathways in *F. rhynchophylla* revealed the top pathways based on the FDR values. The top pathways in *F. rhynchophylla* other than “Biosynthesis of metabolic pathways” and “Metabolic pathways” involved “phenylopropanoid biosynthesis,“ “Photosynthesis–antenna proteins,“ “Starch and sucrose metabolism,” and “Plant hormone signal transduction.“ In *F. chiisanensis*, the top GO terms included protein processing in the ER,” “Starch and sugar metabolism,” “phenylpropanoid biosynthesis,” and “glycolysis and gluconeogenesis.”

### Identification of DEGs in starch and sucrose metabolism

**Fig. 9:**
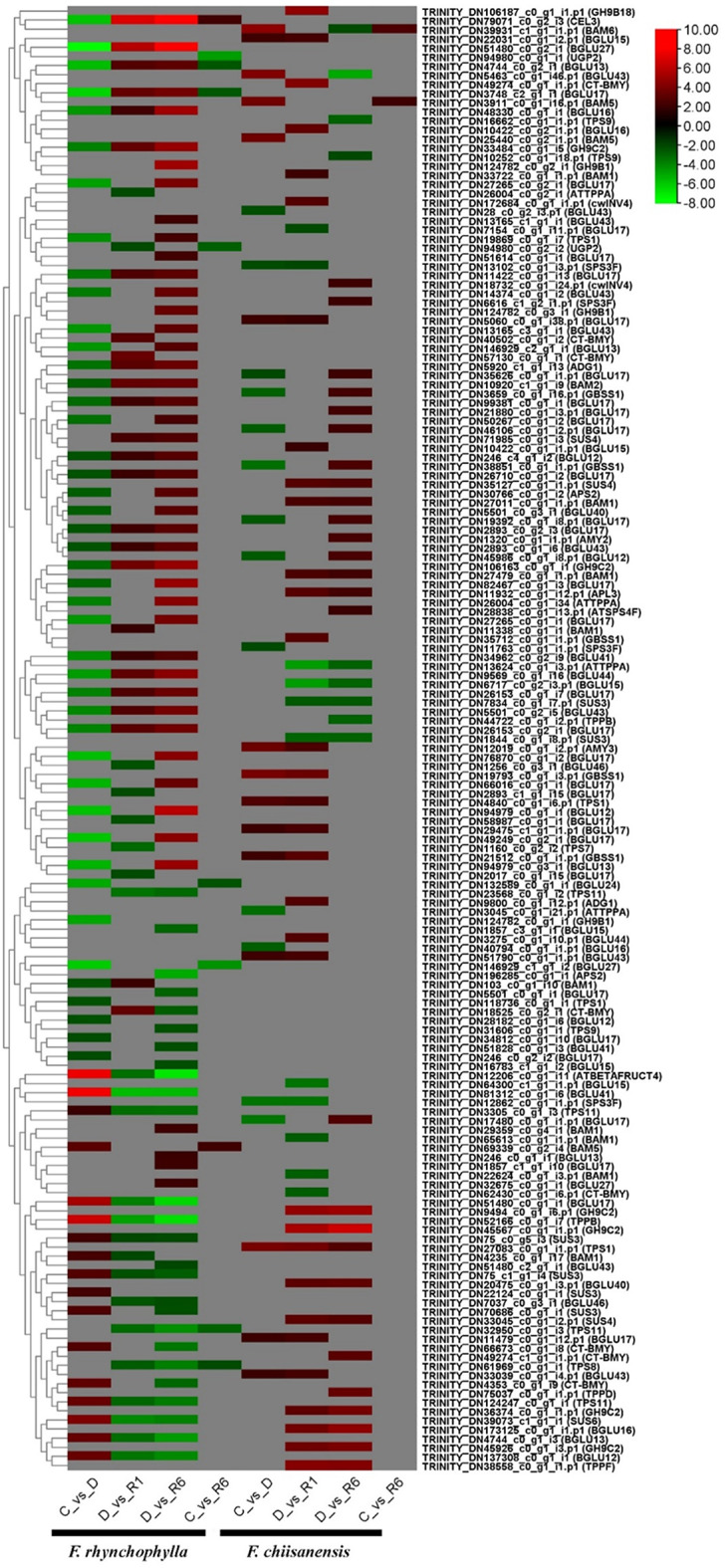
Heatmap representing the expression levels of KEGG pathways, highlighting the expression levels of certain genes involved in starch and sucrose metabolism. The color scale in the upper right corner ranges from the lowest RPKM value indicated in green to the highest RPKM value shown in red

A total of 61, 49, 71, and 10 DEGs were identified in the comparisons of C versus D, D versus R1, D versus R6, and C versus R6 for *F. rhynchophylla*, respectively. It is important to note that only a limited number of differentially expressed genes demonstrated consistent regulation across all comparisons within this species. The major DEGs involved in starch and sucrose metabolism include beta-glucosidases (*BGLU*), cellulases (*CEL*), glycosyl hydrolases (*GH9C*), ADP glucose pyrophosphorylases (*ADG*), leucine-rich repeat receptor-like kinase family genes (*BAM*), trehalose phosphatase/synthase (*TPS*), sucrose-phosphate synthase (*SPS*), haloacid dehalogenase-like hydrolase (*ATTPPA*), alpha-amylase-like protein (*AMY*), cell wall invertase (*cwINV*), UDP-glycosyltransferase superfamily protein, sucrose synthase (*GBSS*), glucose-1-phosphate adenylyltransferase family protein (*APL3*), chloroplast beta-amylase (*CT-BMY*), and trehalose-6-phosphate phosphatase (*TPP*). A limited number of genes that were downregulated in the comparison between groups C and D were upregulated in the comparisons between groups D vs. R1, as well as between D and R6. These genes included *BGLU12*, *BGLU13*, *BGLU16*, *BGLU17*, *BGLU27*, *BGLU41*, *BGLU43*, *BGLU44*, *CEL3*, *GH9C2*, *ADG1*, *BAM1*, and *BAM2*. Furthermore, the analysis of DEGs between the control and the 6-day recovery period revealed downregulation of *BGLU13*, *BGLU17*, *CEL3*, *TPS8*, and *TPS11*. In *F. chiisanensis*, comparative analysis of groups C versus D, D versus R1, D versus R6, and C versus R6 revealed the regulation of 28, 43, 36, and 2 DEGs, respectively. In the C vs. D group, 13 DEGs were upregulated and 15 DEGs were downregulated. The downregulated DEGs were *BGLU12*, *BGLU16*, *BGLU43*, *SPS3F*, *GBSS1*, and *ATTPPA*, whereas genes such as *BGLU15*, *BGLU17*, *BGLU43*, *TPS1*, *AMY3*, beta-amylase 5, and beta-amylase 6 were upregulated. In the D vs. R1 comparison, 43 DEGs were significantly upregulated, of which 32 were upregulated, and 11 were downregulated. The upregulated DEGs included *BGLU15*, *BGLU16*, *BGLU17*, *BGLU40*, *BGLU43*, *BGLU44*, *BAM1*, *TPS1*, *AMY3*, *ADG1*, *cwINV4*, *GBSS1*, *APL3*, *GH9C2*, *GH9B18*, *CT-BMY*, and *TPPF*, whereas few genes, such as *ATTPPA*, *BGLU15*, *BGLU17*, *SPS3F*, *SUS3*, *BAM1*, and *CT-BMY*, were downregulated. A total of 36 DEGs were significantly regulated in the D vs. R6 comparison, in which the upregulated genes (27) included *ATSPS4F*, *SPS3F*, *cwINV4*, *BGLU12*, *BGLU16*, *BGLU17*, *BAM1*, *APL3*, *GBSS1*, *SUS4*, *CT-BMY*, *BGLU40*, *TPPD*, *TPPF*, and *GH9C2*. The downregulated DEGs (9) encoded *BGLU43*, *BGLU15*, *TPS9*, *TPPB*, *SUS3*, and *BAM6*. Finally, in the C vs. R6 comparison, only two DEGs were upregulated, which were related to beta-amylase 5 (*BAM5*) and beta-amylase 6 (*BAM6*) (Supplementary Table 4). A heatmap showing the differential expression of DEGs in the starch and sucrose metabolism pathways is shown in Fig. 9.

### Identification of DEGs in enzymatic and Non-enzymatic antioxidant pathways

Enzymes are crucial for managing abiotic stresses in plants, such as drought, by reducing the bioavailability of ROS. The DEGs associated with antioxidant enzymes across all comparisons include Glutathione S-transferase Phi (*GSTF*), Glutathione S-transferase Tau (*GSTU*), catalase (*CAT*), ascorbate peroxidase (*APX*), glutathione-disulfide reductase (*GR1*), glutathione S-transferase family protein (*GSTL3*), manganese superoxide dismutase (*MSD*), cationic amino acid transporter 2 (*CAT2*), iron superoxide dismutase (*FSD*) and glutathione peroxidase 7 (*GPX7*). In the comparison group (C vs. D), a total of 11 DEGs were identified, with three exhibiting downregulation and eight showing upregulation. The downregulated genes included *GSTF7* and *GSTU7*, while the upregulated genes consisted of *CAT2*, *GSTU7*, *APX5*, *APX2*, *GR1*, and *GSTF11*. In the comparisons of groups D versus R1 and C versus R6, only eight DEGs were differentially regulated. Specifically, in the D versus R1 comparison, six DEGs were downregulated, and two were upregulated. Conversely, in the C versus R6 comparison, two DEGs were downregulated, whereas six were upregulated. The downregulated DEGs in the D versus R1 group included *GSTF9*, *GSTF11*, *APX2*, *GR1*, *GSTU7*, and *CAT2*, whereas the upregulated genes included *GSTU7* and *CAT1*. In the D versus R6 comparison, five out of nine DEGs were downregulated and four DEGs were upregulated. The upregulated genes in the D versus R6 group included *GSTU7*,* CAT3*, and *CAT1*, whereas the downregulated DEGs included *GSTF9*, *GSTU7*, *GSTF11*, *GR1*, and *GSTL3*. In the C versus R6 comparison, the upregulated DEGs were *APX2*, *GSTF3*, *MSD1*, *CAT1*, *CAT2*, and *CAT3*, whereas the downregulated DEGs were involved in the regulation of *GSTF9* and *GSTL3*. In *F. chiisanensis*, 21 DEGs exhibited varying regulation across the various experimental combinations: C compared to D (*n* = 6), D compared to R1 (*n* = 7), D compared to R6 (*n* = 8), and C compared to R6 (*n* = 6).

**Fig. 10:**
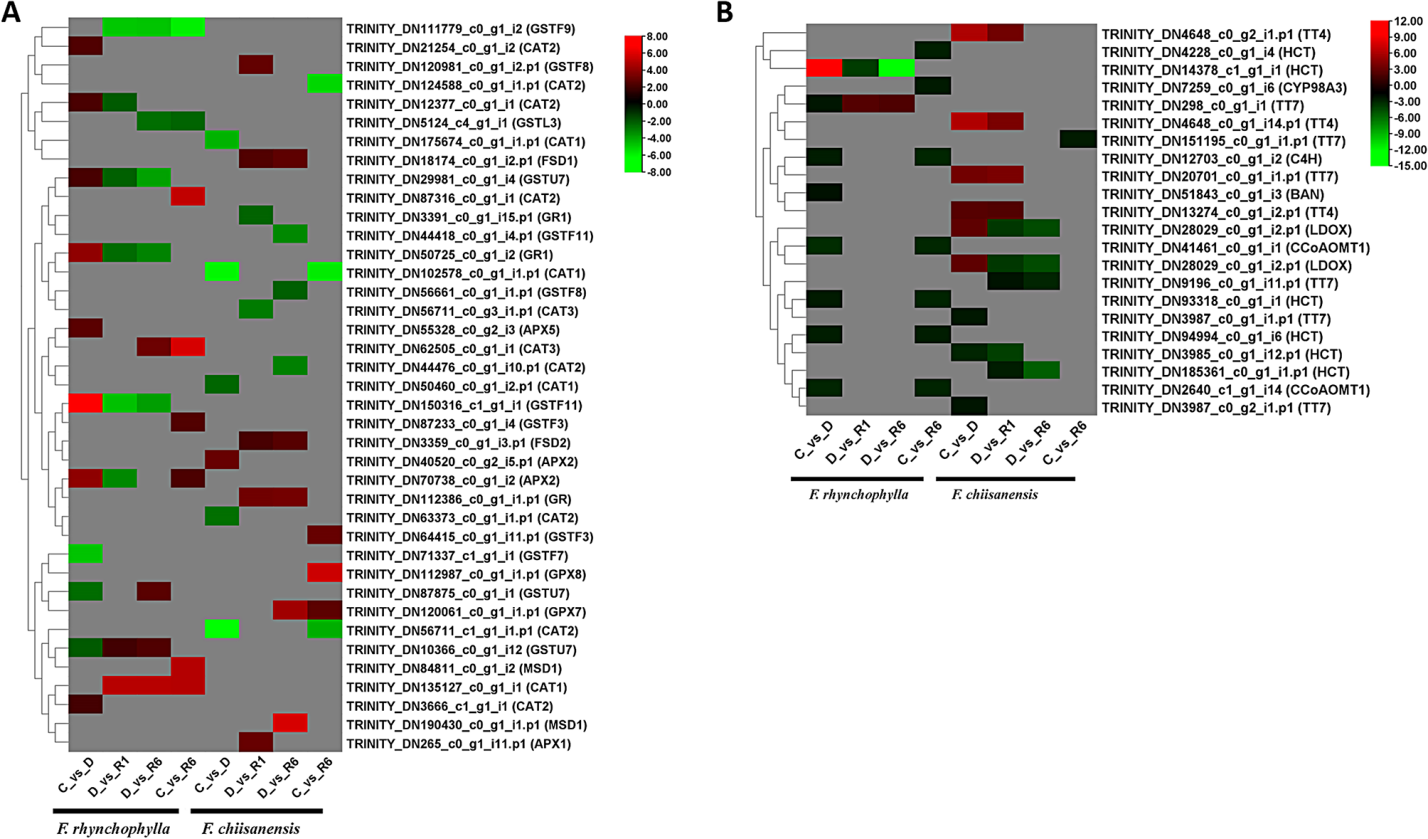
Heatmap representing the expression levels of KEGG pathways, highlighting the expression levels of certain genes involved in (**A**) enzymatic and (**B**) non-enzymatic antioxidant activity. The color scale in the upper right corner ranges from the lowest RPKM value indicated in green to the highest RPKM value shown in red

In the comparison between C and D, five DEGs were found to be downregulated, including catalases (*CAT2*,* CAT1*) and cationic amino acid transporter *(CAT-2*), while only one DEG, *APX2*, which encodes ascorbate peroxidase 2, was upregulated. In the analysis of D versus R1, two DEGs that encode *CAT3* and *GR1* were downregulated, whereas five DEGs, namely *FSD1*, *FSD2*, *GSTF8*, *APX1*, and *GR*, were upregulated. In the D versus R6 comparison, the downregulated DEGs included those encoding *GSTF11*, *CAT2*, and *GSTF8*, whereas the upregulated DEGs comprised *FSD2*, *FSD1*, *GR*, *GPX7*, and *MSD1*. Six DEGs were identified in the C versus R6 group, three of six DEGs were upregulated, specifically those encoding *GPX7*, *GSTF3*, and *GPX8*, and three downregulated DEGs encoding *CAT1* and *CAT2* proteins. A heat map illustrating the differential expression of DEGs within the enzymatic antioxidant pathway is shown in Fig. 10A. Non-enzymatic antioxidant metabolites include flavonoids, phenols, ascorbic acid, and glutathione (*GSH*). The DEGs identified across the non-enzymatic antioxidant reactions included *TT*, *HCT*, *CCoAOMT1*, *C4H*, *HCT*, *CYP98A3*, and *LDOX*. In the case of *F. rhynchophylla*, ten DEGs were identified across the control, drought, and re-watering conditions. In the comparison between control and drought (C vs. D), seven DEGs were downregulated, while one DEG was upregulated. In the comparison of drought to re-watering stages R1 and R6 (D vs. R1 and D vs. R6), two DEGs exhibited differential regulation: one upregulated DEG corresponded to *TT7* and the downregulated DEG was *HCT*. In the C vs. R6 comparison, seven DEGs were down-regulated, including those encoding *CCoAOMT1*, *C4H*, *HCT*, and *CYP98A3*. In *F. chiisanensis*, 12 genes were differentially regulated. In the C vs. D comparison, three DEGs were downregulated, whereas six DEGs were upregulated, including *HCT* and *TT7*. The D vs. R1 comparison revealed nine differentially regulated genes, with five downregulated and four upregulated. The downregulated DEGs included *HCT*, *LDOX*, and *TT7*, whereas the upregulated DEGs included *TT4* and *TT7*. In the D vs. R6 comparison, four DEGs were downregulated and one DEG was downregulated in the C vs. R6 comparison (Supplementary Tables 5 and 6). A heat map illustrating the differential expression of DEGs within the non-enzymatic antioxidant pathway is shown in Fig. 10B.

### Identification of DEGs in plant hormone signaling

**Fig. 11:**
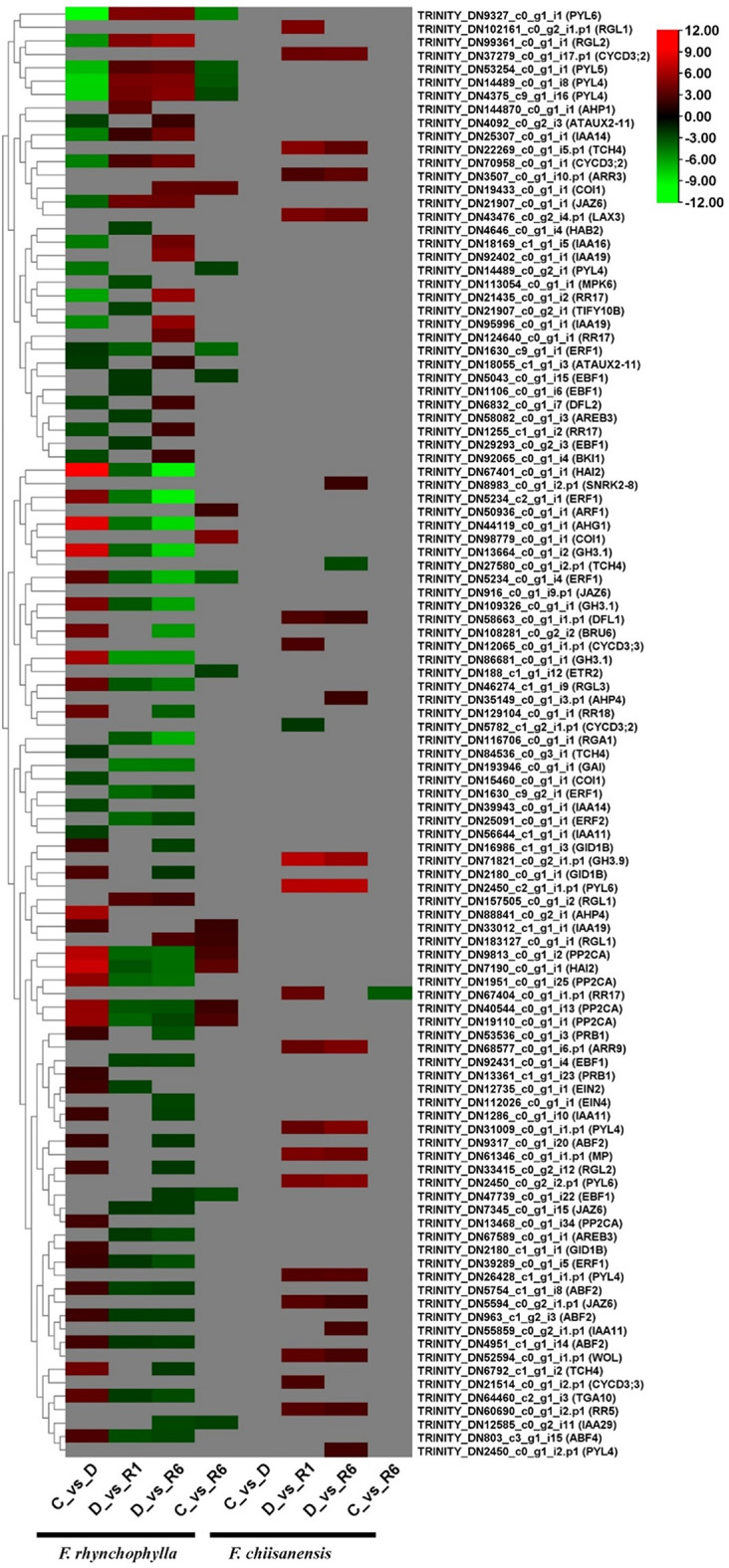
Heatmap representing the expression levels of KEGG pathways, highlighting the expression levels of certain genes involved in plant hormone signaling. The color scale in the upper right corner ranges from the lowest RPKM value indicated in green to the highest RPKM value shown in red

Plant hormones are crucial signaling molecules that regulate key aspects of growth and development and how plants respond to environmental stress. Abiotic stresses, such as drought, salinity, extreme temperatures, and flooding, significantly affect plant growth and survival. To adapt to and withstand these challenges, plants rely on complex sensing, signaling, and stress response systems. We examined the regulation of several genes involved in plant hormone signaling in both the *Fraxinus* species. These include the PYR1-like protein (*PYL*), response regulator (*RR*), Arabidopsis response regulator (*ARR*), AUX/IAA transcriptional regulator family protein (*ATAUX*), indole-3-acetic acid inducible (*IAA*), protein phosphatase 2CA (*PP2CA*), auxin-responsive GH3 family protein (*GH3.1*), ethylene response factor (*ERF*), abscisic acid responsive elements-binding factor (*ABF*), alpha/beta-Hydrolases superfamily protein (*GID1B*), CYCLIN D3 (*CYCD3*), auxin-responsive GH3 gene homologue (*DFL1*), jasmonate-zim-domain protein (*JAZ*), CHASE domain-containing histidine kinase protein (*WOL*), like AUX1 (*LAX*), transcription factor B3 family protein/auxin-responsive factor AUX/IAA-like protein (*MP*), RGA-like (*RGL*), putative indole-3-acetic acid-amido synthetase (*GH3.9*), xyloglucan endotransglucosylase/hydrolase family protein (*TCH4*), protein kinase superfamily protein (*SNRK2-8*), and HPT phosphotransmitter (*AHP*). A total of 82 DEGs were identified in *F. rhyncophylla* and 26 DEGs were identified in *F. chiisanensis*. Of the 82 DEGs in *F. rhyncophylla*, 56, 45, 59, and 20 were affected in the C vs. D, D vs. R1 comparison, 59 in the D vs. R6, and 20 in the C vs. R6 comparisons, respectively. The number of DEGs in *F. rhyncophylla* was significantly higher than in *F. chiisanensis*. In the comparison between C and D for *F. rhynchophylla*, the key DEGs included *PYL4*, *RR17*, *ATAUX2-11*, and *IAA1*4. In contrast, the DEGs that were significantly downregulated were *PP2CA*,* GH3.1*, *ERF1*, *ABF4*, and *GID1B*. The analysis indicated that the DEGs that were upregulated in the comparison between C and D exhibited downregulation in the D versus R1 and D versus R6 comparisons; conversely, the opposite trend was observed. The primary proteins encoded by DEGs in the C versus R6 comparison demonstrated both upregulation and downregulation in a consistent manner under control and drought conditions. As previously mentioned, only a small number of genes were influenced by plant hormone signaling pathways. In *F. chiianensis*, a total of 26 DEGs were identified in *F. chiianensis*. In the C vs. D comparison, no DEGs were differentially expressed. However, in the D versus R1 comparison, one DEG, *CYCD3;2*, was downregulated, whereas several others were upregulated. The up-regulated DEGs included *CYCD3*,* 2*, *CYCD3*,* 3*, *ARR3*, *DFL1*, *RR5*, *PYL4*, *JAZ6*,* WOL*, *RR17*, *ARR9*, *LAX3*, *MP*, *RGL1*, *PYL6*, and *GH3.9*. In the comparison between D and R6, only one DEG (*TCH4*) was downregulated, whereas the following DEGs were upregulated: *SNRK2-8*, *AHP4*, *DFL1*, *PYL4*, *JAZ6*, *IAA11*, *RR5*, *WOL*, *TCH4*, *ARR3*, *LAX3*, *CYCD3;2*, *MP*, *ARR9*, *PYL6*, and *GH3.9*. In the C versus R6 comparison, only one DEG (*RR17*) was found to be downregulated (Supplementary Table 7). A heatmap illustrating the differential expression of DEGs in the plant hormone signaling pathway is shown in Fig. 11.

### Identification of DEGs in proline signaling

Proline metabolism is increasingly recognized as a crucial factor influencing cellular signaling pathways that can either promote apoptosis or enhance cell survival. Studies have shown that Proline metabolism impacts these signaling pathways by increasing the generation of ROS within the mitochondria via the electron transport chain. Additionally, we investigated the DEGs associated with proline metabolism in *F. rhynchophylla* and *F. chiisanensis*. A greater number of DEGs were identified in *F. rhynchophylla* than in *F. chiisanensis*, with 15 DEGs being regulated across all comparison groups in *F. rhynchophylla*. In the C vs. D comparison, 11 DEGs were upregulated and none were downregulated. Eight DEGs were significantly upregulated, which corresponded to the following proteins: *ASP3*, *PAO4*, *ALDH7B4*, *ADC*, *ALDH3F1*, *ASP3*, and *P5CS1*. In the analysis of comparative group D versus R1, it was noted that all DEGs associated with *P5CS1*, *ADC1*, and *ADC2* were significantly downregulated. Similarly, 11 DEGs were downregulated in D versus R6, encoding *ALDH3F1*, *ADC1*, *ADC2*, *ASP3*, *P5CS1*, and *PAO4*. Only three DEGs were differentially regulated in the C vs. R6 comparison, encoding *ADC1* and *ADC2*.

**Fig. 12:**
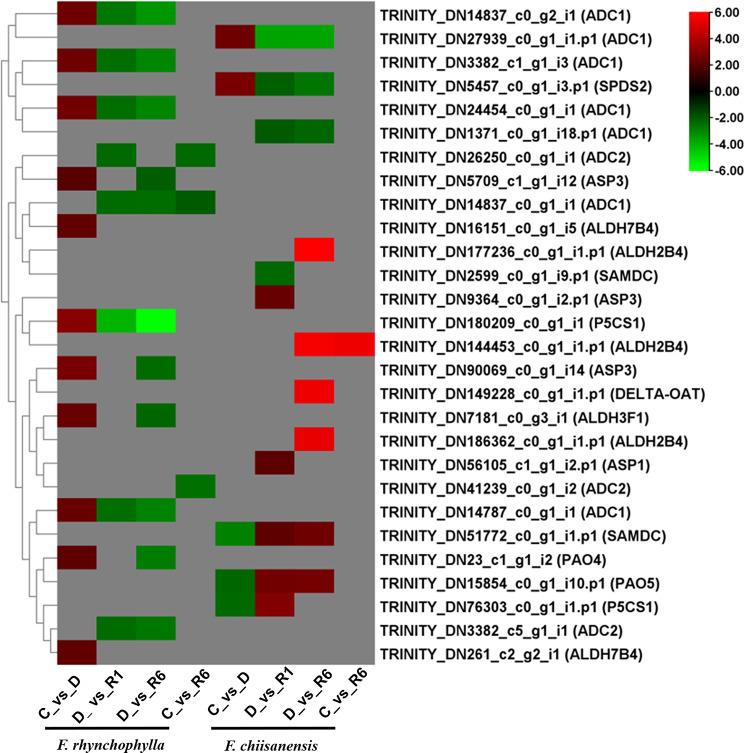
Heatmap representing the expression levels of KEGG pathways, highlighting the expression levels of certain genes involved in proline signaling. The color scale in the upper right corner ranges from the lowest RPKM value indicated in green to the highest RPKM value shown in red

DEGs in *F. chiisanensis* exhibited varied regulation across all comparison groups. In the comparison between C and D, only five DEGs were identified, with *SAMDC*,* P5CS1*, and *PAO5* showing significant downregulation, whereas *ADC1* and *SPDS2* were upregulated. In the D versus R1 comparison, four out of nine DEGs were downregulated, and five were upregulated. Specifically, *ADC1*, *SAMDC*, and *SPDS2* were down-regulated, whereas *ASP1*, *SAMDC*, *ASP3*, *PAO5*, and *P5CS1* were up-regulated. In the D versus R6 comparison, DEGs such as *PAO5*, *ALDH2B4*, *DELTA-OAT*, and *SAMD*C were markedly upregulated, whereas *ADC1* and *SPDS2* were downregulated. In comparison between C and R6, only one DEG, *ALDH2B4*, was upregulated (Supplementary Table 8). A heatmap illustrating the differential expression of DEGs within the proline metabolism pathway is shown in Fig. 12.

We have delineated several pathways associated with drought stress in plants, particularly in trees. Through KEGG pathway enrichment analysis, we identified four distinct pathways implicated in drought stress responses. These pathways encompass Starch and Sucrose Metabolism, Enzymatic and Non-enzymatic Antioxidant Pathways, Plant Hormone Signaling, and Proline Metabolism. Starch and sucrose metabolism serve as critical regulators during periods of drought stress in plants. Several enzymes, including ADP glucose pyrophosphorylase 1, alpha amylase, beta glucosidase, beta-amylase, sucrose synthase, and trehalose 6-phosphate synthase, collaborate with trehalose-6-phosphatase/synthase to facilitate the accumulation of soluble sucrose while reducing starch levels. Within the enzymatic antioxidant pathway, two key enzymes, catalase and glutathione S-transferase, are essential. Catalase is particularly significant, playing a crucial role in mitigating various abiotic stresses, including drought. In the non-enzymatic antioxidant pathway, cytochrome P450 emerges as a pivotal component in enhancing drought stress tolerance in trees. Notably, we observed an increased expression of the cytochrome P450 enzyme *TT7* in both *Fraxinus* species, especially under drought conditions. Additionally, we recognized the regulation of proline metabolism in *Fraxinus*, involving two enzymes: Aldehyde dehydrogenase (*ALDH*) and arginine decarboxylase (*ADC*) responsible for the accumulation of proline in plant cells.

### Co-expression network construction and identification of WGCNA modules

**Fig. 13:**
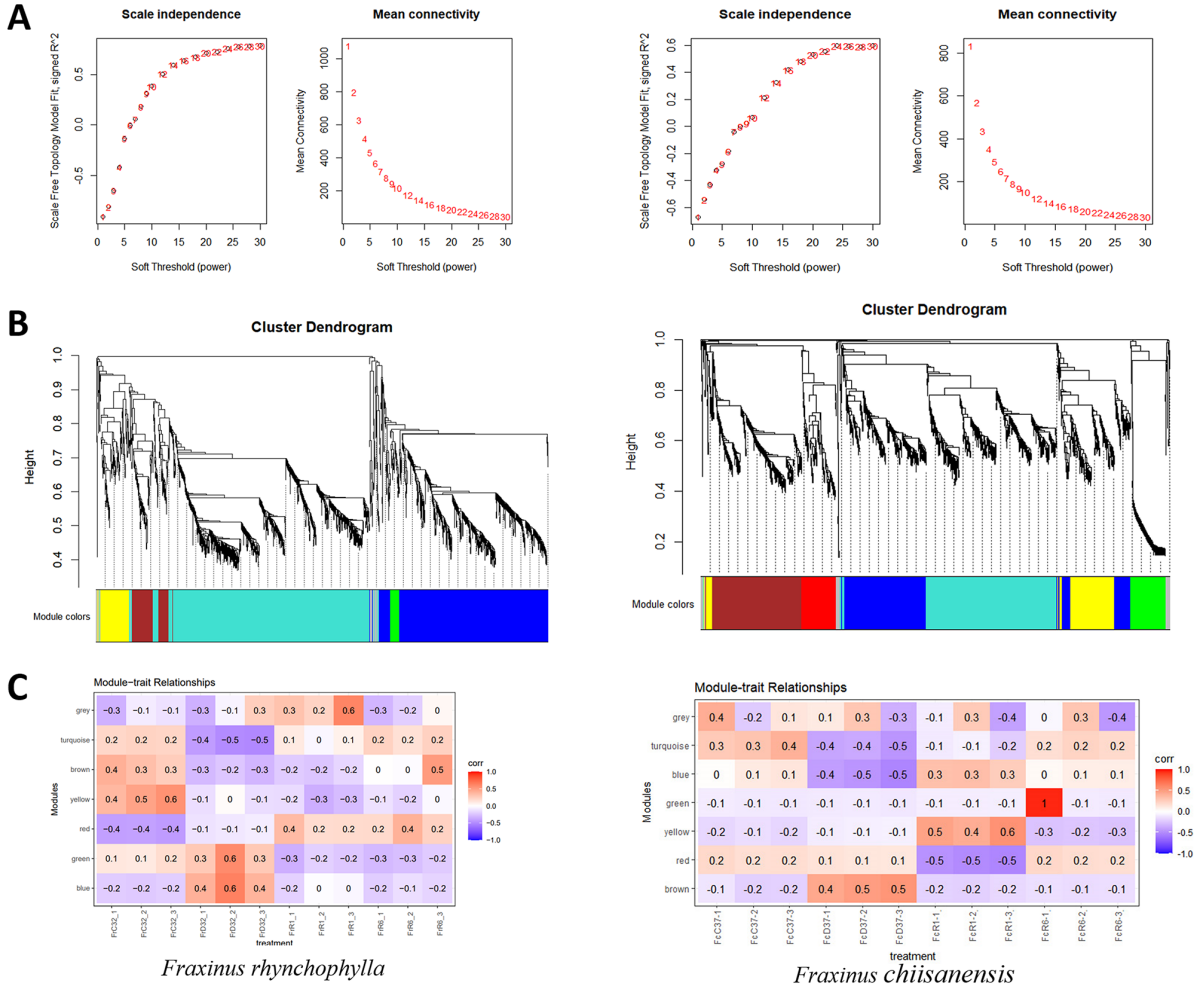
Analysis of module-trait correlations and the WGCNA co-expression network generated. (**A**) The summary of network indices (Y-axis) is plotted against the soft-thresholding power (X-axis). The values indicated in the plot correspond to the respective soft-thresholding powers. (**B**) The co-expression modules were identified through the Dynamic Tree Cut method of the hierarchical clustering tree. Each leaf, represented by a small vertical line, corresponds to a gene. The branches of the dendrogram are color-coded to reflect the degree of relatedness among genes, facilitating the formation of modules. Genes that demonstrate high co-expression levels (correlation > 0.5) were aggregated into a single module, resulting in a total of four distinct modules. (**C**) Correlation between WGCNA modules and physiological traits. Each row corresponds to a module, while the columns represent drought-related characteristics. The color of each cell indicates the correlation coefficient between the traits and the respective module. A positive correlation is denoted in red, whereas a negative correlation is indicated in blue. The correlation coefficient is displayed as the top number in each cell, with the P-value presented in parentheses below

Gene co-expression network analysis involves the clustering of genes and delineating modules that exhibit similar expression patterns organized along distinct branches. Each branch symbolizes a co-expression module, with various colors denoting different modules. During the Weighted Gene Co-expression Network Analysis (WGCNA), over half of the samples were excluded, leading to the examination of the expression patterns of 34,465 genes from a total of 50,681 from *F. rhynchophylla* and 33,443 genes out of 50,674 genes from *F. chiisanensis* obtained from transcriptome sequencing. We successfully identified seven distinct modules (Fig. 13) in *F. rhynchophylla* and *F. chiisanensis* based on the similarities in their expression patterns. These modules were linked to varying levels of drought stress, and a module-trait relationship was established. Notably, in *F. rhynchophylla*, genes within the “blue” (596 genes) and “green” (40 genes) modules exhibited a positive correlation with drought stress, while those in the “red” (37 genes) and “yellow” (123 genes) modules showed a negative correlation. In *F. chiisanensis*, the “brown” (324 genes) module showed a positive correlation to drought stress. In comparison, those in the “turquoise” (482 genes) and “blue” (391 genes) modules were negatively correlated, regardless of drought conditions.

### Validation of the RNA-seq results using qPCR

**Fig. 14:**
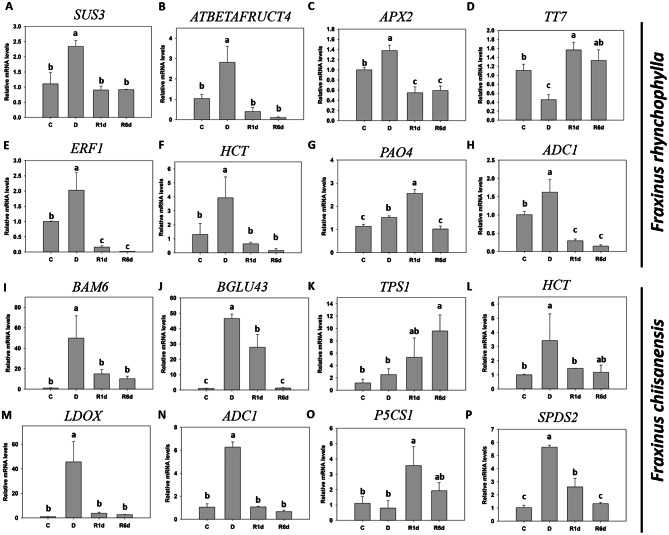
Validation of the differential expression of 16 genes using qPCR. (**A**-**H**) qPCR results for *F. rhynchophylla* genes and (**I**-**P**) *F. chiisanensis*. (**A**) sucrose synthase 3 (*SUS3*). (**B**) Glycosyl hydrolases family 32 protein (*ATBETAFRUCT4*). (**C**) ascorbate peroxidase 2 (*APX2*). (**D**) Cytochrome P450 superfamily protein (*TT7*). (**E**) ethylene responsive factor (*ERF1*). (**F**) hydroxycinnamoyl transferase (*HCT*). (**G**) polyamine oxidase 4 (*PAO4*). (**H**) arginine decarboxylase 1 (*ADC1*). (**I**) beta-amylase 6 (*BAM6*). (**J**) beta glucosidase (*BGLU43*). (**K**) trehalose-6-phosphate synthase (*TPS1*). (**L**) hydroxycinnamoyl-CoA shikimate/quinate hydroxycinnamoyl transferase (*HCT*). (**M**) leucoanthocyanidin dioxygenase (*LDOX*). (**N**) arginine decarboxylase 1 (*ADC1*). (**O**) delta1-pyrroline-5-carboxylate synthase 1 (*P5CS1*). (**P**) spermidine synthase 2 (*SPDS2*). Different lowercase letters indicate significant differences (ANOVA with Tukey’s honestly significant difference test, *p* < 0.05)

qPCR was performed to confirm the RNA-seq results (Fig. 14). In *F. rhynchophylla*, the expression levels of *SUS3*, *ATBETAFRUCT4*, and *HCT* increased during drought and returned to normal levels after water resupply. The expression levels of *APX2*, *ADC1*, and *ERF1* increased during drought and then decreased to a level lower than the previously normal level during the recovery period. In contrast, *TT7* expression decreased during drought conditions and increased when water was resupplied. The expression level of *PAO4* increased after drought, peaked at the R1 stage, and then decreased at the R6d stage. In *F. chiisanensis*, the expression levels of *BAM6*, *BGLU43*, *HCT*, *LDOX*, *ADC1*, and *SPDS* increased during drought conditions and tended to recover to normal levels when water was resupplied. The expression level of *TPS1* increased significantly on R6d. In addition, the expression levels of *P5CS1* increased significantly on R1d and tended to normalize to the previous level or decrease on R6d. The log_2_ fold-change values obtained from the RNA-seq expression analysis strongly correlated with the qPCR results, as indicated by an R^2^ value of 0.7147 (Supplementary Fig. 5). In *F. rhyncophylla*, quantitative PCR analysis revealed positive results for several genes, including TRINITY_DN12206_c0_g1_i11 (*ATBETAFRUCT4*), TRINITY_DN14378_c1_g1_i1 (*HCT*), TRINITY_DN14837_c0_g2_i1 (*ADC*1), and TRINITY_DN5234_c2_g1_i1 (ERF1), all of which are situated within the ‘blue’ module and exhibit a positive correlation with drought stress (Supplementary Table 9). Conversely, in *F. chiianensis*, the gene TRINITY_DN28029_c0_g1_i2.p1 (*LDOX*) was identified in the brown module, while TRINITY_DN39931_c1_g1_i1.p1 (*BAM6*) and TRINITY_DN5463_c0_g1_i46.p1 (*BGLU43*) were located in the yellow module correlating with the drought stress.

## Discussion

### Impact of drought stress on phenotypic traits

When evaluating the impact of drought stress on *F. rhynchophylla* and *F. chiisanensis*, notable differences emerged in various physiological responses. Both species exhibited distinct responses to drought stress, with *F. rhynchophylla* showing greater temperature and growth reduction than *F. chiisanensis*. *F. rhynchophylla* exhibited resilience, with moderate temperature and substantial RWC recovery after re-watering. In contrast, *F. chiisanensis* displayed delayed recovery of certain physiological parameters, unique temperature dynamics, and limited RWC recovery. *F. chiisanensis* showed less temperature change and higher RWC than *F. rhynchophylla* under drought stress conditions. However, even during the recovery period after water resupply, its RWC and plant temperature did not recover immediately. The continued decrease in RWC and temperature, even after water resupply, suggests that *F. chiisanensis* needs a longer recovery time to properly normalize its moisture content and temperature. The RWC in the leaf is intricately linked to cell volume and offers a nuanced perspective on the equilibrium between water supply and transpiration rate. This indicator is pivotal for plant recovery from stress as it influences both yield and stability. In particular, under drought stress, the RWC is deemed more informative than other water potential parameters. Typically, RWC values range from 98% in turgid and transpiring leaves to approximately 40% in severely desiccated leaves [58]. These results led us to assume that *F. chiisanensis* actively regulates plant temperature via more active transpiration. These findings highlight species-specific variations in the ability to cope with and rebound from drought-induced stresses.

### Photosynthetic efficiency and chlorophyll content

Chlorophyll fluorescence analysis revealed differences in the drought stress responses of *F. rhynchophylla* and *F. chiisanensis*. *F. rhynchophylla* exhibited an earlier decline and subsequent recovery in Fv/Fm levels, whereas *F. chiisanensis* exhibited a delayed response. The parameter Fv/Fo represents the ratio of the photochemical to non-photochemical de-excitation fluxes of excited chlorophyll. Changes in this ratio suggest alterations in the rate of electron transport from PSII to primary electron acceptors with respect to their density and size [59]. The Fv/Fm represents the maximum yield of primary photochemistry and the overall PSII photosynthetic capacity. A decrease in the Fv/Fm value below 0.8 indicates inactivation or damage to the PSII reaction core. Therefore, the observed reduction in the Fv/Fm in both ash species implies damage to the photosynthetic system due to drought stress. The chlorophyll content increased in both *F. rhynchophylla* and *F. chiisanensis* under drought stress, with a decrease in the chlorophyll a/b ratio. After water resupply, *F. rhynchophylla* maintained higher chlorophyll levels than the control, while *F. chiisanensis* showed a reduction, in some cases below the control levels. The chlorophyll content is an effective indicator of desiccation tolerance, and a reduction in leaf chlorophyll is a common trait under drought stress. This decrease is often associated with oxidative stress and chlorophyll damage, which affect photosynthetic activity. However, certain plants, such as canola, cypress, and sesame, may experience an initial increase in chlorophyll content, followed by stabilization [60–63]. This aligns with the findings of the present study, which observed an increase in chlorophyll a, chlorophyll b, and total chlorophyll content during drought treatment. Carotenoid regulation is species- and stress intensity-dependent. Although mild drought stress often leads to a decrease in carotenoid content, severe stress may result in a slight increase [64, 65]. In this study, both ash species subjected to drought treatment exhibited increased carotenoid content, indicating a severe drought impact on seedlings. These results indicate species-specific variations in chlorophyll dynamics and adaptation strategies for drought stress.

### Metabolite changes and osmotic adjustment

We investigated the regulation of soluble sugars, starches, soluble proteins, and proline in response to drought stress in *F. rhynchophylla* and *F. chiisanensis*. By comprehensively comparing these results, we confirmed that *F. rhynchophylla* and *F. chiisanensis* have different drought stress and recovery response mechanisms. In particular, *F. chiisanensis* showed better recovery of fructose, and in the case of sucrose, *F. chiisanensis* showed no significant response, whereas *F. rhynchophylla* increased significantly; *F. rhynchophylla* showed better accumulation of starch during recovery. For water-soluble proteins, *F. rhynchophylla* and *F. chiisanensis* showed different reactivities. Protein concentrations increased during the *F. rhynchophylla* recovery period, and that of *F. chiisanensis* increased significantly in response to drought, but recovered to previous levels after re-watering. This also suggests that there are differences in the osmolyte regulatory sensitivity and defense mechanisms of the two species against drought stress, and it can be inferred that there are differences in the composition and activity of the proteins involved in drought stress and recovery. Sucrose and starch are known to act as osmoregulators in response to drought stress. By observing the state transition of sucrose and starch, it is an indicator that can confirm whether the plant is in a state of water shortage and whether it is responding appropriately to osmoregulation. Both species exhibited increased soluble sugar content and decreased starch content when subjected to drought stress. However, there was a significant difference in the degree of response. In *F. rhynchophylla*, sucrose levels increased greatly, and starch levels decreased only slightly, whereas, in *F. chiisanensis*, starch content decreased greatly, but sucrose levels only increased relatively little.

Among the DEGs related to sucrose and starch, the gene that showed the most active changes was the β-glucosidase (*BGLU*) gene, which was identified in both species. There were 52 DEGs in *F. rhynchophylla* and 23 DEGs in *F. chiisanensis*, and the changes in their expression were complex. The fact that so many *BGLU*-related DEGs responded suggests that both species try to overcome drought through the regulation of soluble sugar content, and we were able to confirm that they were organically linked at the genetic level to the measured changes in soluble sugar and starch contents. Sugars stored in plants can be broken down into various complexes by BGLUs, which helps maintain the stability of plant physiological processes [66]. Plants synthesize secondary glycogroup metabolites to manage abiotic stress. Glycosylation increases solubility and stability. The BGLU family catalyzes this process, aiding cell membrane stability, osmotic balance, cell wall degradation, lignification, and signal transduction [67]. Starch can be broken down into smaller carbohydrate molecules by the catalytic action of β-amylase (BAM) [68]. Six BAM-related DEGs were identified in *F. rhynchophylla* and eight in *F. chiisanensis*, showing complex responses to drought and water resupply. It is also thought that these factors affect the regulation of changes in soluble sugar and starch content during drought and recovery. Cell wall invertase (cwINV) breaks down sucrose into glucose and fructose in the extracellular matrix and plays key roles in the distribution of assimilates and sugar signaling [69]. *cwINV4* levels increased only during the recovery period of *F. chiisanensis*, which may have influenced the dramatic regulation of glucose and fructose content during this period. *SUS* was confirmed to be in DEG in both species. In *F. rhynchophylla*, *SUS3* and *SUS6* expression increased during recovery after showing decreased expression in drought; only *SUS4* showed an increased expression after a decrease. In *F. chiisanensis*, *SUS3* expression decreased and *SUS4* expression increased during the recovery period. Proline levels increased significantly and then recovered in response to drought in both species, showing more dramatic responses and changes in *F. rhynchophylla* than in *F. chiisanensis*. *P5CS* (Δ1-pyrroline-5-carboxylate synthase) is the central rate-limiting enzyme gene for proline synthesis. It catalyzes the NADPH-dependent reduction of glutamate to γ-glutamate-semialdehyde (GSA) [70]. Proline-related DEGs exhibited completely different patterns between the two tree species: those in *F. rhynchophylla* increased during drought and then decreased during recovery, whereas those in *F. chiisanensis* showed the opposite trend. Arginine acts as a substitute precursor for glutamate during proline biosynthesis. The levels of *ADC* (arginine decarboxylase) increased significantly during drought in both tree species and then recovered when water was resupplied [71]. A more significant change was observed in *F. rhynchophylla*, consistent with the results of our proline content analysis. ASP (aspartate aminotransferase) is responsible for converting aspartate to glutamate, the main precursor of proline. Our transcriptome analysis showed differential *ASP* expression in the two tree species [72]. *ASP* expression in *F. rhynchophylla* increased under drought conditions and decreased in R6d. However, its expression in *F. chiisanensis* showed differences in response time and treatment, which increased after re-watering. OAT is a key enzyme involved in proline biosynthesis. The expression of *DELTA-OAT* increased significantly only in D versus R6 for *F. chiisanensis*. These results showed a temporal difference from the actual measured changes in proline content. It has been reported that DELTA-OAT is important for arginine catabolism but is not essential for proline biosynthesis [73]. Collectively, these findings underscore the species-specific responses to drought stress, revealing dynamic adjustments in the metabolic and osmotic regulatory mechanisms that contribute to adaptive strategies in *F. rhynchophylla* and *F. chiisanensis*.

### Antioxidant enzymes and oxidative damage

By examining the antioxidant responses, MDA levels, and H_2_O_2_ concentrations in *F. rhynchophylla* and *F. chiisanensis* under drought stress and recovery conditions, their adaptation mechanisms can be indirectly inferred (Fig. 3). In *F. rhynchophylla*, elevated MDA levels and H_2_O_2_ concentrations during drought stress suggested oxidative stress, which was corroborated by the modulation of antioxidant enzyme expression. The decrease in SOD levels upon re-watering may indicate a regulatory role in the mitigation of excessive ROS. The significant increase in APX activity post-water resupply reflects active ROS scavenging (H_2_O_2_), while the delayed recovery of POD suggests sustained oxidative pressure. In *F. chiisanensis*, the sustained elevation of MDA and H_2_O_2_ levels post-water resupply indicated a prolonged impact of drought stress. The rapid recovery of SOD activity and the significant increase in the later stages imply a dynamic antioxidant response, possibly to combat persistent ROS. APX recovery to normal levels and CAT fluctuation post-resupply suggest a coordinated effort in ROS detoxification, while unchanged POD indicates nuanced regulation. Overall, these findings highlight species-specific strategies for managing oxidative stress during drought and recovery, emphasizing the importance of antioxidant enzymes and signaling molecules, such as MDA and H_2_O_2_, in understanding plant adaptation to environmental challenges.

Fe-SOD is sensitive to H_2_O_2_ concentrations; the *FSD1* and *FSD2* genes were identified in this transcriptome analysis [74]. However, it has been reported that MSD1-related Mn-SOD does not respond to H_2_O_2_ concentrations. MSD1 was found to be upregulated in *F. rhynchophylla* in C vs. R6 and *F. chiisanensis* in D vs. R6, demonstrating differences in the timing of the DEG response (Fig. 10A). In both species, H_2_O_2_ levels increased and decreased with drought and water resupply; however, *FSD* gene expression increased during the recovery treatment only in *F. chiisanensis*. Moreover, there was a difference in the timing of the increase and decrease in the expression of these SOD-related DEGs compared to the actual SOD level. The expression of CAT-related DEGs changed in all treatment groups for both tree species. However, there was a large difference in the expression change propensity. For example, the expression of all DEGs increased in *F. rhynchophylla* in D vs. R1, except TRINITY_DN12377_c0_g1_i1 (*CAT2*). In contrast, all CAT-related DEGs were downregulated in *F. chiisanensis*. These DEGs are presumed to have influenced the increase in SOD levels during drought in *F. rhynchophylla* or the tendency for SOD levels to be maintained or slightly decreased during recovery in *F. chiisanensis*. The expression of all APX-related DEGs increased upon drought treatment; however, APX activity decreased, resulting in conflicting results. This was presumed to be due to the need for a time difference in protein expression or to induce enzyme expression to compensate for the low level of APX activity.

Plant GSTs are grouped into ten different classes: tau (GSTU), phi (GSTF), lambda (GSTL), and dehydroascorbate reductase (DHAR). These isoenzymes and many other GSTs exhibit glutathione peroxidase (GPX) activity and can convert lipid peroxides and other peroxides into less harmful compounds [75]. GPXs are non-heme peroxidases that catalyze the reduction of H_2_O_2_ or organic hydroperoxides to water or the corresponding alcohols using glutathione (GSH) or thioredoxin (TRX) as reducing agents [76]. GR uses NADPH to promote the reduction of GSH, which is involved in metabolic regulation and antioxidant processes [77]. It has also been reported to improve tolerance to drought stress tolerance [78]. For *GST* genes (*GSTF*, *GSTU*, and *GSTL*), many DEGs were observed in *F. rhynchophylla*, and almost all of them showed increased and then decreased expression during drought and recovery (*GSTF9*, *GSTL3*, *GSTU7*, and *GSTF11*). The rest (*GSTF3*, *GSTF7*, and *GSTU7*) showed the opposite pattern. In *F. chiisanensis*, increased (*GSTF8* and *GSTF3*) or decreased (*GSTF11* and *GSTF8*) expression levels were observed during recovery. *GR* and *GPX* expression in *F. rhynchophylla* increased in drought and decreased in recovery (TRINITY_DN50725_c0_g1_i2 (*GR1*)). However, in *F. chiisanensis*, DEGs were identified only in recovery (TRINITY_DN3391_c0_g1_i15.p1 (*GR1*), TRINITY_DN112386_c0_g1_i1.p1 (*GR*), TRINITY_DN112987_c0_g1_i1.p1 (*GPX8*), and TRINITY_DN120061_c0_g1_i1.p1 (*GPX7*)), confirming that there is a difference in expression time in these genes compared to those in *F. chiisanensis*. Leucoanthocyanidin dioxygenase (LDOX) is an anthocyanidin synthase that converts leucoanthocyanidins into anthocyanins [79]. The expression level of *LDOX* in *F. chiisanensis* increased under drought conditions and then decreased upon water resupply, consistent with the results of our anthocyanin content analysis. TT4 is a chalcone and stilbene synthase family protein that plays a central role in anthocyanin biosynthesis. Here, especially in *F. chiisanensis*, its expression increased very strongly in the C vs. D and C vs. R1 comparisons, which is also thought to have influenced the increase in anthocyanin content. C4H (cinnamate-4-hydrolase) is a cytochrome P450 superfamily protein that plays an important catalytic role in converting phenylalanine into coumarin coenzyme A and regulating anthocyanin synthesis [80]. In our analysis, the DEGs in *F. rhynchophylla* decreased under drought conditions and did not recover until R6d. This was also presumed to have affected the anthocyanin content.

### Hormonal regulation under drought stress

Measurements of ABA and IAA, which are important hormones in drought stress responses, provide clues to the adaptive strategies of *F. rhynchophylla* and *F. chiisanensis* (Fig. 4). In *F. rhynchophylla*, a significant increase in ABA levels under drought conditions suggests its role in triggering stress responses. Remarkably, ABA levels remain elevated even after re-watering until R6d, indicating a prolonged influence on plant physiology. The decrease in IAA level during drought stress and its subsequent recovery, especially at the R6d stage, implies a regulatory role in growth recovery during the water resupply period. In *F. chiisanensis*, the substantial increase in ABA level during drought stress and its continued elevation post-water resupply suggest a persistent signaling mechanism that is potentially linked to prolonged stress perception. The slight decrease in ABA level at R6d indicates gradual recovery. The significant reduction in IAA level during drought and its subsequent recovery, surpassing the control levels in R6d, indicate its involvement in restoring plant growth and development. Overall, the contrasting patterns of ABA and IAA levels in the two species revealed distinct hormonal regulation strategies during drought stress and recovery, underscoring the nuanced roles played by these hormones in orchestrating adaptive responses.

In addition to its important role in plant growth and development, IAA has recently been shown to interact with other hormones to enhance drought tolerance under drought stress [81]. The TIFY (TIF(F/Y)XG) protein plays a central role in the jasmonic acid signaling pathway and plays an important role in plant development, defense, and stress responses [82]. Among the DEGs of *F. rhynchophylla*, *TIFY10B* expression decreased in D vs. R1. PYR/PYL/RCAR receptors that recognize ABA transduce ABA-responsive signals by inhibiting protein kinase type 2 C protein phosphates (PP2Cs) and activating (SNF1)-related protein kinase 2 (SnRK2) [83]. In *F. rhynchophylla*, *PYL4*, *PYL5*, and *PYL6* expression decreased during drought and increased after water resupply but still maintained increased levels compared to the control. In *F. chiisanensis*, *PYL4* and *PYL6* expression increased during the recovery period. Considering these results, including our observation that ABA content was still increased even on R6d, it was presumed that it was for the feedback control of receptors on ABA concentration. In addition, in *F. rhynchophylla*, the expression of ABA-related DEGs (*HAI2* (highly_ABA-induced_PP2C_protein_2), *ABF2* (abscisic_acid_responsive_elements-binding_factor_2), *AREB* (ABA-responsive_element_binding_protein_3), and *AHG1* (protein _phosphatase_2C_family_protein)) tended to increase during drought and decrease during recovery.

IAA-related DEGs were identified in both tree species. In *F. rhynchophylla*, *IAA14* and *IAA16* expression decreased during drought and increased during recovery. However, *IAA11* levels increased during drought. In *F. chiisanensis*, only the expression of *IAA11* increased in D compared to R6. In addition, auxin-related DEGs include *ATAUX2-11* (AUX/IAA_transcriptional_regulator_family_protein), *ARF1* (auxin_response_factor_1), *BRU6* (auxin-responsive_GH3_family_protein), *GH3.1* (auxin-responsive_GH3_family_protein), and *DFL2* (auxin-responsive_GH3_family_protein) in *F. rhynchophylla* and *LAX3* (like AUX1), *DFL1* (auxin-responsive GH3 family protein), and *GH3.9* (putative indole-3-acetic acid-amido synthetase GH3.9) in *F. chiisanensis*. These changes in expression are thought to have closely influenced the decline in IAA content during drought and subsequent recovery. In particular, the increase in the expression levels of *IAA19* and *ARF1*, which are known to affect plant growth in D vs. R6d in *F. rhynchophylla*, indicated that the plant escaped drought stress to some extent and recovered to a normal state [84].

### Summary of the response of two *Fraxinus* species to drought stress and Re-Watering

When comparing the response to drought stress and water resupply, *F. rhynchophylla* showed a more immediate and sensitive response, while *F. chiisanensis* showed a milder and more delayed response. *F. rhynchophylla* showed more severe growth retardation and temperature increase, and its chlorophyll fluorescence response decreased more quickly. In addition, significant increases in stress indices were also observed. However, most of these recovered quickly after the water supply. In contrast, *F. chiisanensis* showed less growth retardation, and the decline in the chlorophyll fluorescence response due to drought was slower. Its stress indices increased relatively weakly, but most of the time, it did not recover to previous levels, even after water was supplied. The reactivity of antioxidant enzymes also differed between the two species. Antioxidant enzymes such as SOD, CAT, and APX showed a more appropriate response and recovery in *F. rhynchophylla*; those in *F. chiisanensis* showed a weak drought stress response, showing reduced activities and later recoveries. GO term analysis revealed that photosynthesis and enzymatic activity were the most responsive components during drought and recovery. Moreover, KEGG pathway analysis identified pathways related to “starch and sucrose metabolism” and “phenylpropanoid biosynthesis” in both species.

In both species, drought stress and recovery-related DEGs showed greater changes in *F. rhynchophylla* than in *F. chiisanensis*. The upregulated DEGs in C vs. D showed a notable difference, and the downregulated DEGs in D vs. R1 and D vs. R6 also displayed significant variations between the two species. These findings suggest that *F. rhynchophylla* and *F. chiisanensis* respond differently to drought and recovery. When examining the 20 DEGs that responded most significantly, *F. rhynchophylla* responded to drought more sensitively than *F. chiisanensis*. Although they showed similar numbers of DEGs in the recovery phase, the expression of other DEG-related genes showed different patterns (Fig. 5A and B). In particular, most of the DEGs in C vs. R6 were new DEGs unrelated to drought or recovery responses, suggesting that both species escaped the effects of drought to some extent and entered the next stage of gene regulation. The DEG involved in both drought and recovery in *F. rhynchophylla* was *LAX3*, which acts as an auxin influx carrier (Fig. 5C) [85]. Two DEGs were expressed only during the recovery period in *F. rhynchophylla*: *CEL3*, which encodes cellulase 3 and is involved in the immune response, and *HSFA6B*, which is a positive regulator downstream of ABA signaling and is necessary for plants to secure heat tolerance to drought [86, 87]. In *F. chiisanensis*, two DEGs were involved in drought and recovery responses (Fig. 5D). *GAPCP-1* is known to play an important role in root development and sugar and amino acid balance [88]. *AT3G56230* is presumed to be a BTB/POZ domain-containing protein and a protein-protein interaction motif involved in transcriptional regulation, cytoskeletal dynamics, ion channel assembly and gating, and protein targeting for ubiquitination. In the recovery period, the DEG *AT3G47850* is a putative tRNA(Ile)-lysidine synthetase, which is involved in isoleucine synthesis, promotes JA-Ile formation and affects the regulation of plant growth and defense against various stress signals [89, 90]. Another one is MAP kinase kinase 5 (*MKK5*), which regulates plant immunity, growth, and development [91]. Across both species, several genes were also commonly involved in the drought and recovery responses, such as *BGLU* and *TPS* in sugar metabolism, *CAT* and *GSTF* in antioxidant activity, *PYL4* and *RR17* in hormonal regulation, and *ADC1* and *ASP3* in proline synthesis, illustrating shared and distinct genetic responses in managing drought and recovery. Collectively, these findings highlight the unique adaptations and stress-regulation mechanisms of both species. Due to differences in drought tolerance between the two *Fraxinus* species, the sampling periods were adjusted to capture their respective stress responses, which may complicate direct comparisons. Starch and sucrose metabolism and antioxidant pathways are key metabolic pathways that regulate drought stress responses in both species. *F. rhynchophylla* exhibits faster and more pronounced changes in these pathways, indicating an efficient drought tolerance mechanism. In contrast, *F. chiisanensis* shows slower responses, which may lead to slower recovery and limited resilience to prolonged drought. This analysis emphasizes the importance of these metabolic pathways in regulating drought tolerance. Future studies should aim to identify conserved genes and pathways involved in drought tolerance and further investigate the interplay between physiological and genetic responses. Such efforts will enhance our understanding of shared and unique mechanisms underlying drought resilience in these species (Supplementary Fig. 6).

The differences in drought tolerance between *F. rhynchophylla* and *F. chiisanensis* were attributed to hormonal regulation, osmotic adjustment via water-soluble sugars, and the ability to maintain SOD activity. In *F. rhynchophylla*, the genes *BGLU* and *ATBETAFRUCT*, which were upregulated under drought stress, influenced the regulation of intracellular water-soluble sugars. Additionally, the reduced auxin concentration during drought stress increased the expression of the *TPPB* gene, thereby modulating trehalose levels and contributing to osmotic adjustment [92]. Drought stress also elevated ABA levels, which in turn upregulated the expression of *HAI2* and *PP2CA*, facilitating signal transduction for drought stress responses [93]. Furthermore, the increased expression of MSD1 under drought conditions enhanced SOD activity, leading to a reduction in ROS.

In *F. chiisanensis*, the IAA- and ABA-related genes, including *GH3.9* and *PYL6*, exhibited increased expression under drought stress, indicating their involvement in maintaining hormonal homeostasis [81, 94]. During recovery, the upregulation of *MSD1* further enhanced SOD activity, contributing to ROS scavenging. Additionally, the increased expression of *TT4* under drought conditions led to anthocyanin accumulation, which provided enhanced protection against ROS-induced damage [95]. Furthermore, drought stress induced the expression of *GH9C2*, which facilitated cell wall polysaccharide metabolism, secondary metabolism, and signal transduction, thereby strengthening drought tolerance mechanisms [96].

## Conclusion

As inferred from their habitats, *F. rhynchophylla* exhibited a more phenotypically sensitive response to drought than *F. chiisanensis*, and its physiological response changed rapidly and significantly. Additionally, a considerable number of DEGs were identified during drought and recovery. Nevertheless, *F. rhynchophylla* showed a very rapid recovery after water resupply. In contrast, *F. chiisanensis* showed drought-resistant characteristics with little change in plant temperature, and its physiological change was relatively weak; however, several parameters did not recover well to the previous levels. Its DEG changes were also less responsive to drought. This study is the first to investigate the physiological and transcriptomic changes in response to drought stress and recovery in two species of *Fraxinus*. Although this study had to account for differences in drought treatment durations and the sample collection periods of some samples between the two species, it enabled a comprehensive comparison of their drought stress and recovery responses. Our results confirmed the possibility of investigating drought tolerance through changes in several physiological indicators and gene expression profiles, and they are expected to be used as a reference for related studies. To refine these results, the common pathway TFs for these genes should be identified. Overall, these results will allow us to infer the potential causes of drought tolerance differences between *F. rhynchophylla* and *F. chiisanensis* and will be helpful for selecting and developing drought-resistant varieties.

## Electronic supplementary material

Below is the link to the electronic supplementary material.

Supplementary Material 1

Supplementary Material 2

Supplementary Material 3

## Data Availability

The raw data were deposited in the NCBI Short Read Archive database under the accession number PRJNA1172350.
