# Supplementary material for: Comparative phenotypic, physiological, and transcriptomic responses to drought and recovery in two Fraxinus species
Source: BMC Plant Biol. 2025 Mar 18;25:348. doi: 10.1186/s12870-025-06372-6 (PMC11916329; doi:10.1186/s12870-025-06372-6)

Supplementary Table 1. Sequence of primers used for qPCR in of *Fraxinus rhynchophylla* and *Fraxinus chiisanensis.*

| **Gene** | **Forward primer (5’→3’)** | **Reverse primer (5’→3’)** |
| --- | --- | --- |
| *FrATBETAFRUCT4* | TTCGAGCTGCTGGTGACTTT | CCTCCTACTCTCCCATCCCC |
| *FrSUS3* | ACCACCAGTATCAGGCAAGC | GCTGCAAGGGTTCTGGAGAT |
| *FrAPX2* | GCTCCTCAACACCTCCAACA | CCTGTCTTTCGCCCTCTTGT |
| *FrHCT* | TCATCTTTGCCAAGCCTCCC | GCACCCCTCCCACAATAACA |
| *FrTT7* | TGACCGGGTTATAGGCCAGA | CACAGTTCGGGGTCTCTTCC |
| *FrADC1* | GCAAGTTCCTGTCAGTCTCCA | CAAAGGCCATCAACTGCAGC |
| *FrPAO4* | CTTGTTCTCATTGCCCACGC | AAGGTTGAGGACGGGAGGAA |
| *FrERF1* | GCACCCAAATCCTCAAACACC | CGGTGGAAAGGGTGAGAGAG |
| *FcBAM6* | GGTGTCAACAAAAGCGGTCC | ACGGGAATGGCTTGATTGGT |
| *FcBGLU43* | GAGGAGAAGCTCGTGAAAGGA | GAACCGGCCCTTACACCAAT |
| *FcTPS1* | GCTATGAGTTTGTGGCCTGC | TCAGCAGGCATATTCAAAGCA |
| *FcHCT* | TAGTCCGCTCCCTTTGCATG | TTGAGCTTGGCAAGAAACGC |
| *FcLDOX* | CACCTCGTCAACCATGGGAT | TCCTGCCACTCAAGTTGACC |
| *FcADC1* | GCTGCAGTTCACGGTGAGAA | ACATGGTAAGTGCGGACAGG |
| *FcSPDS* | TTGGAGGAGGAGATGGTGGT | GTGTTACACGGGGATCCTCA |
| *FcP5CS1* | AATGAATGGGTCCCGCTCAC | TCATATCCTGCCTGCTGTGC |
| *Frax Actin* | TCCTCTTCCAGCCTTCTTT | TTCCTTGCTCATACGGTCA |
| *Frax 18SrRNA* | GATGGTGTGCCCAGATTATTGC | GAAGACCTAAGCCCTGACCCAT |

| Fraxinus Species | Sample Name | Read count | Data in GB | Chemistry | Barcode used | Adapter trimmed count | Read loss after trimming | Data loss in % | Alignment in Reference Genome |
| --- | --- | --- | --- | --- | --- | --- | --- | --- | --- |
| *Fraxinus rhynchophylla* | FrC32-1 | 33454775 | 6.75786455 | 101*2 | TCTGTTGG+CCATTCGA | 31491472 | 1963303 | 5.868528484 | 71.49% |
|  | FrC32-2 | 33520897 | 6.771221194 | 101*2 | TATCGCAC+ACACTAAG | 32169511 | 1351386 | 4.031473263 | 71.70% |
|  | FrC32-3 | 37047412 | 7.483577224 | 101*2 | CGCTATGT+GTGTCGGA | 35637656 | 1409756 | 3.805275251 | 72.18% |
|  | FrD32-1 | 35439247 | 7.158727894 | 101*2 | AGGTTATA+CGGAACTG | 34230001 | 1209246 | 3.412166178 | 70.18% |
|  | FrD32-2 | 31950352 | 6.453971104 | 101*2 | GAACCGCG+TAAGGTCA | 30592091 | 1358261 | 4.251161302 | 69.45% |
|  | FrD32-3 | 31744903 | 6.412470406 | 101*2 | CTCACCAA+TTGCCTAG | 30442322 | 1302581 | 4.103276044 | 69.52% |
|  | FrR1-1 | 33345649 | 6.735821098 | 101*2 | GTATGTTC+TTCCTGTT | 31975111 | 1370538 | 4.110095443 | 72.32% |
|  | FrR1-2 | 33480445 | 6.76304989 | 101*2 | ACGCACCT+CCTTCACC | 31717875 | 1762570 | 5.264476025 | 71.62% |
|  | FrR1-3 | 24152082 | 4.878720564 | 101*2 | TACTCATA+GCCACAGG | 23177521 | 974561 | 4.035101405 | 72.94% |
|  | FrR6-1 | 30138516 | 6.087980232 | 101*2 | CGTCTGCG+ATTGTGAA | 28938673 | 1199843 | 3.981095154 | 72.43% |
|  | FrR6-2 | 32551268 | 6.575356136 | 101*2 | TCGATATC+ACTCGTGT | 31336103 | 1215165 | 3.733080383 | 72.74% |
| *Fraxinus chiianensis* | FrR6-3 | 32260083 | 6.516536766 | 101*2 | CTAGCGCT+GTCTACAC | 30801762 | 1458321 | 4.520512238 | 72.77% |
|  | Fc_C37-1 | 32809818 | 6.627583236 | 101*2 | AACTGTAG+TGCGGCGT | 31915148 | 894670 | 2.726836217 | 80.36% |
|  | Fc_C37-2 | 29907046 | 6.041223292 | 101*2 | GGTCACGA+CATAATAC | 28930617 | 976429 | 3.264879453 | 77.51% |
|  | Fc_C37-3 | 29082938 | 5.874753476 | 101*2 | CTGCTTCC+GATCTATC | 27798299 | 1284639 | 4.417156891 | 79.06% |
|  | Fc_D37-1 | 33367004 | 6.740134808 | 101*2 | TCATCCTT+AGCTCGCT | 31863141 | 1503863 | 4.507036352 | 70.92% |
|  | Fc_D37-2 | 29854174 | 6.030543148 | 101*2 | AGGTTATA+CGGAACTG | 28758006 | 1096168 | 3.671741178 | 75.36% |
|  | Fc_D37-3 | 26156753 | 5.283664106 | 101*2 | GAACCGCG+TAAGGTCA | 24719708 | 1437045 | 5.493973201 | 75.76% |
|  | Fc_R1-1 | 37462259 | 7.567376318 | 101*2 | CTCACCAA+TTGCCTAG | 36499494 | 962765 | 2.569959809 | 77.53% |
|  | Fc_R1-2 | 35169154 | 7.104169108 | 101*2 | TCTGTTGG+CCATTCGA | 34185304 | 983850 | 2.79747986 | 75.41% |
|  | Fc_R1-3 | 37377615 | 7.55027823 | 101*2 | TATCGCAC+ACACTAAG | 36502054 | 875561 | 2.342474232 | 75.88% |
|  | Fc_R6-1 | 30823665 | 6.22638033 | 101*2 | CGCTATGT+GTGTCGGA | 29577853 | 1245812 | 4.04173871 | 76.05% |
|  | Fc_R6-2 | 27059817 | 5.466083034 | 101*2 | GTATGTTC+TTCCTGTT | 25786127 | 1273690 | 4.706942401 | 74.71% |
|  | Fc_R6-3 | 32970954 | 6.660132708 | 101*2 | ACGCACCT+CCTTCACC | 31829303 | 1141651 | 3.462596199 | 77.55% |

Supplementary Table 2. RNA seq statistics of de novo assembly *F. rhyncophylla* and *F. chiianensis.*

Supplementary Table 3. DEG Information

|  | Comparison | All DEG | Up(logFC) | Down(logFC) | Up(logFC,pval) | Down(logFC,pval) |
| --- | --- | --- | --- | --- | --- | --- |
| *Fraxinus rhynchophylla* | C vs D | 50566 | 3898 | 4845 | 2600 | 3448 |
|  | D vs R1 | 50262 | 3339 | 3855 | 2140 | 2333 |
|  | D vs R6 | 50365 | 4487 | 3652 | 3035 | 2330 |
|  | C vs R6 | 50170 | 1751 | 1972 | 587 | 730 |
| *Fraxinus chiianensis* | C vs D | 50125 | 2333 | 4074 | 1077 | 2837 |
|  | D vs R1 | 50104 | 3430 | 2515 | 2376 | 1394 |
|  | D vs R6 | 50518 | 4166 | 1895 | 2384 | 904 |
|  | C vs R6 | 50527 | 2330 | 1307 | 475 | 346 |

Supplementary Figure 1. Mapping Percentage


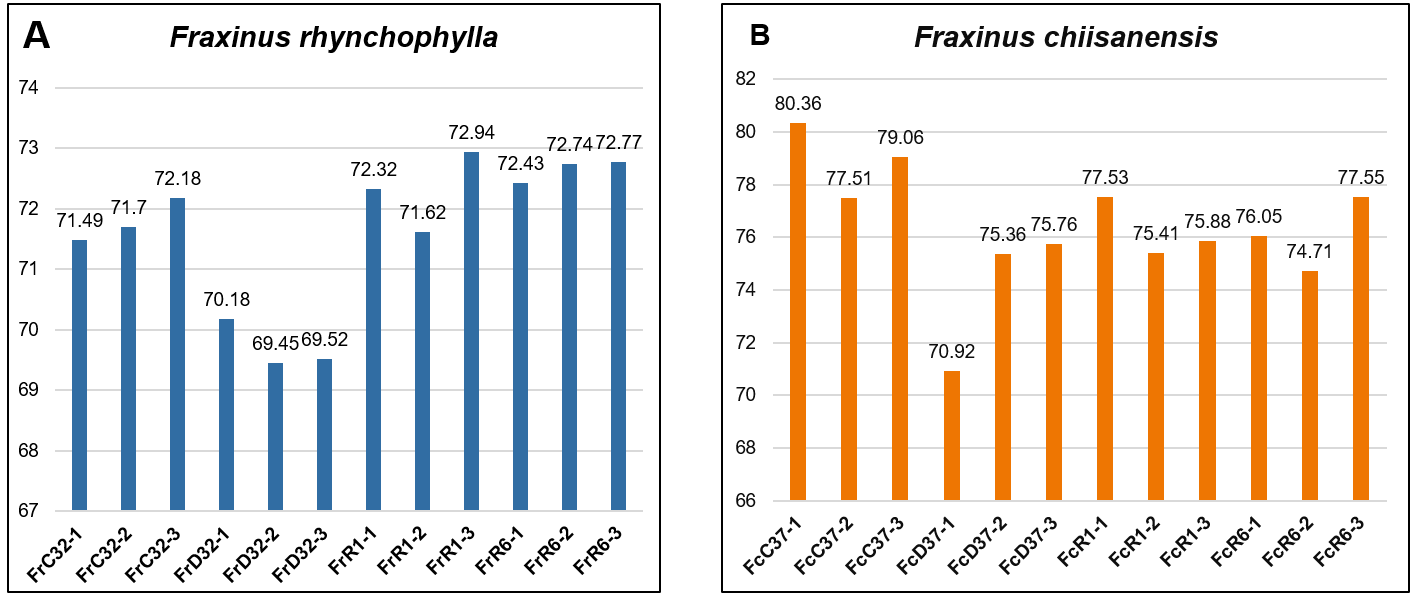


Supplementary Figure 2. PCA Plot


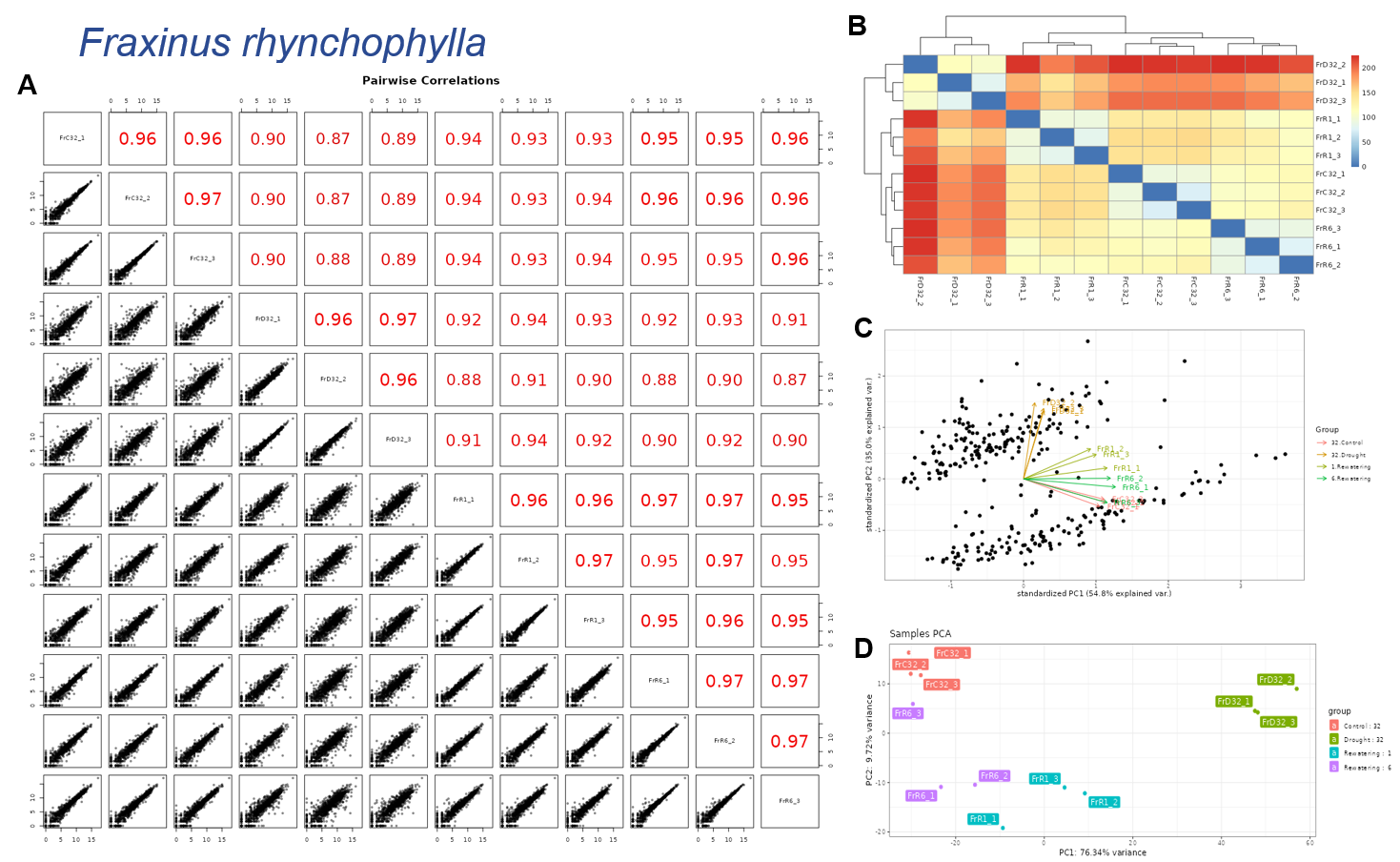


Supplementary Figure 2. Analysis of RNA-Seq Data from Various Libraries. (A) Correlograms depict the correlation score matrix for all pairwise libraries at each time point. (B) A heatmap illustrates the principal component analysis (PCA) of expression in R cells across four distinct time points. A scatterplot comparison of biological replicates is presented based on (C) genes and (D) samples, with PC1 representing the first principal component and PC2 representing the second principal component. The R value indicates the Pearson correlation coefficient.

Supplementary Figure 3. PCA Plot


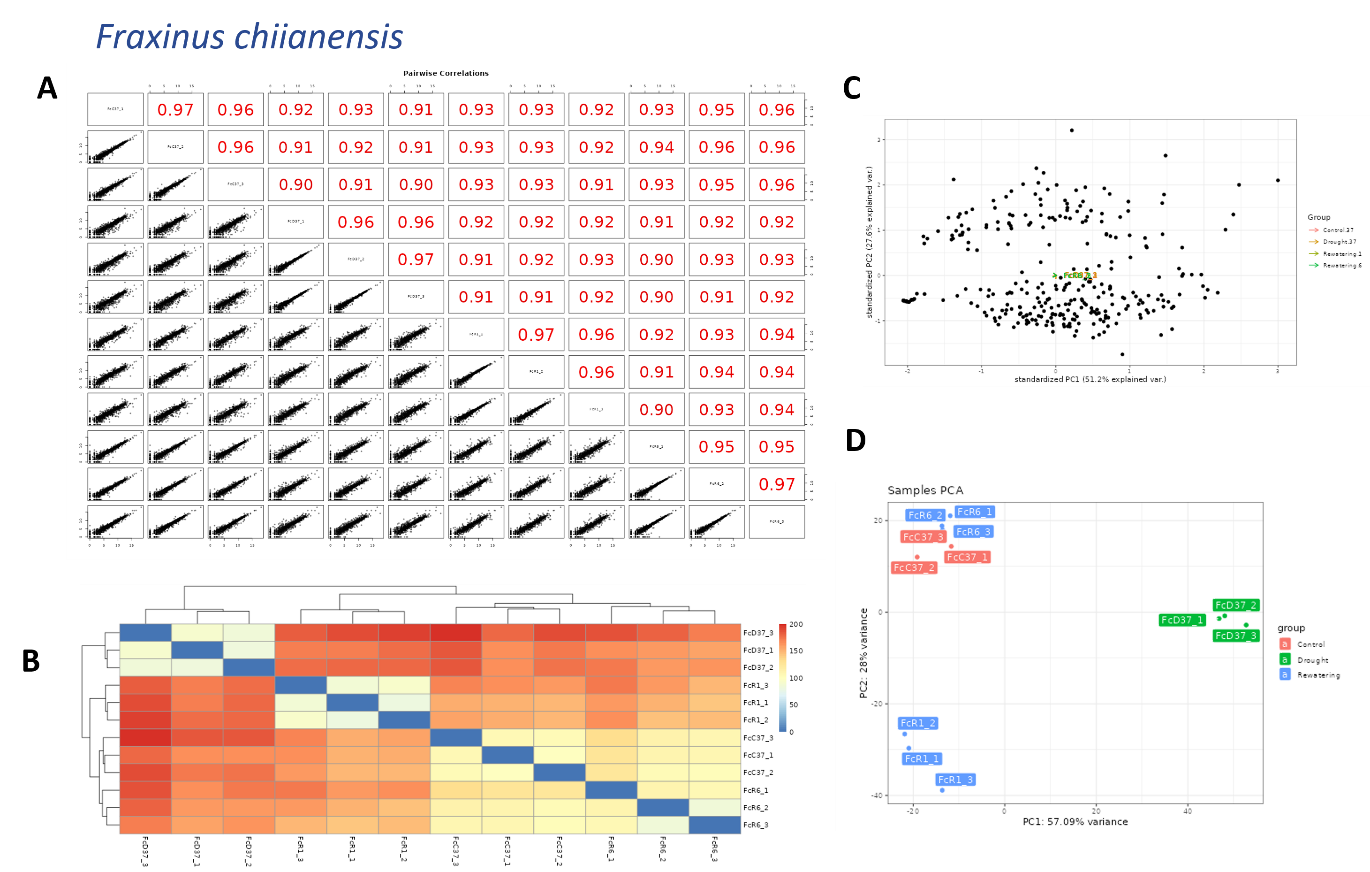


Supplementary Figure 3. Analysis of RNA-Seq Data from Various Libraries. (A) Correlograms depict the correlation score matrix for all pairwise libraries at each time point. (B) A heatmap illustrates the principal component analysis (PCA) of expression in R cells across four distinct time points. A scatterplot comparison of biological replicates is presented based on (C) genes and (D) samples, with PC1 representing the first principal component and PC2 representing the second principal component. The R value indicates the Pearson correlation coefficient.

Supplementary Figure 4. Volcano Plot – *Fraxinus rhynchophylla* and *Fraxinus chiisanensis*


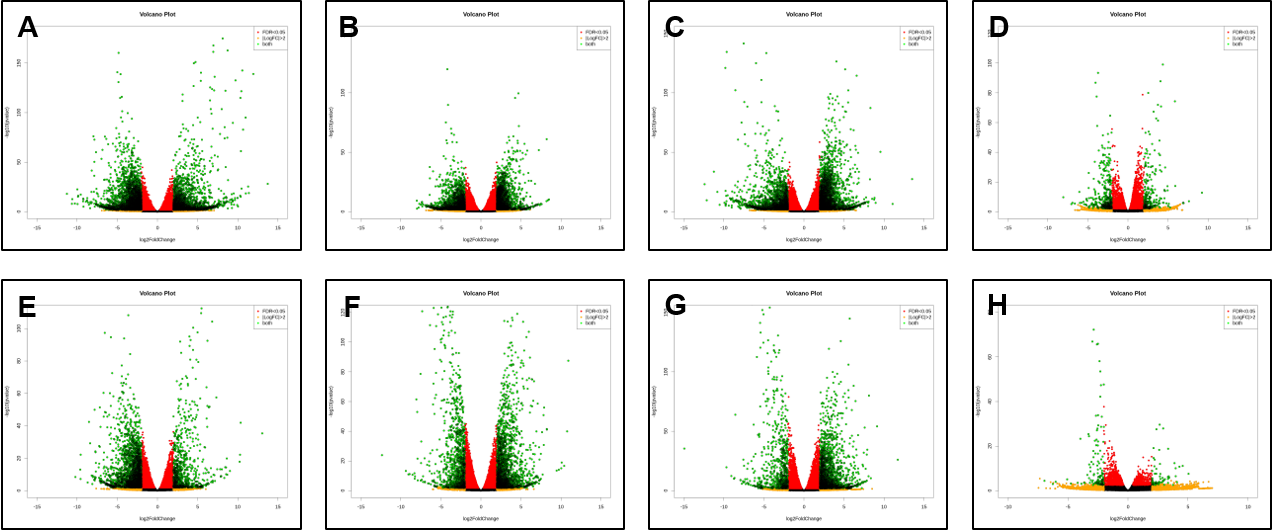


Supplementary Figure 4. The volcano plot illustrates the RNA-seq data for *F. rhynchophylla* (A-D) and *F. chiisanensis* (E-H). It provides a schematic overview of various comparisons for *F. rhynchophylla*, including C vs D (A), D vs R1 (B), D vs R6 (C), and C vs R6 (D). Similarly, it depicts the comparisons for *F. chiisanensis*, namely C vs D (E), D vs R1 (F), D vs R6 (G), and C vs R6 (H). Genes that are upregulated and downregulated are indicated by green and red dots, respectively, while genes that are not differentially expressed are shown as orange dots.

Supplementary Figure 5. Linear regression analysis of fold change of the gene expression between RNA-SEQ and qPCR.

Supplementary Figure 6. Comparison of *F.rhynchophylla* and *F.chiisanensis* response to drought stress and recovery


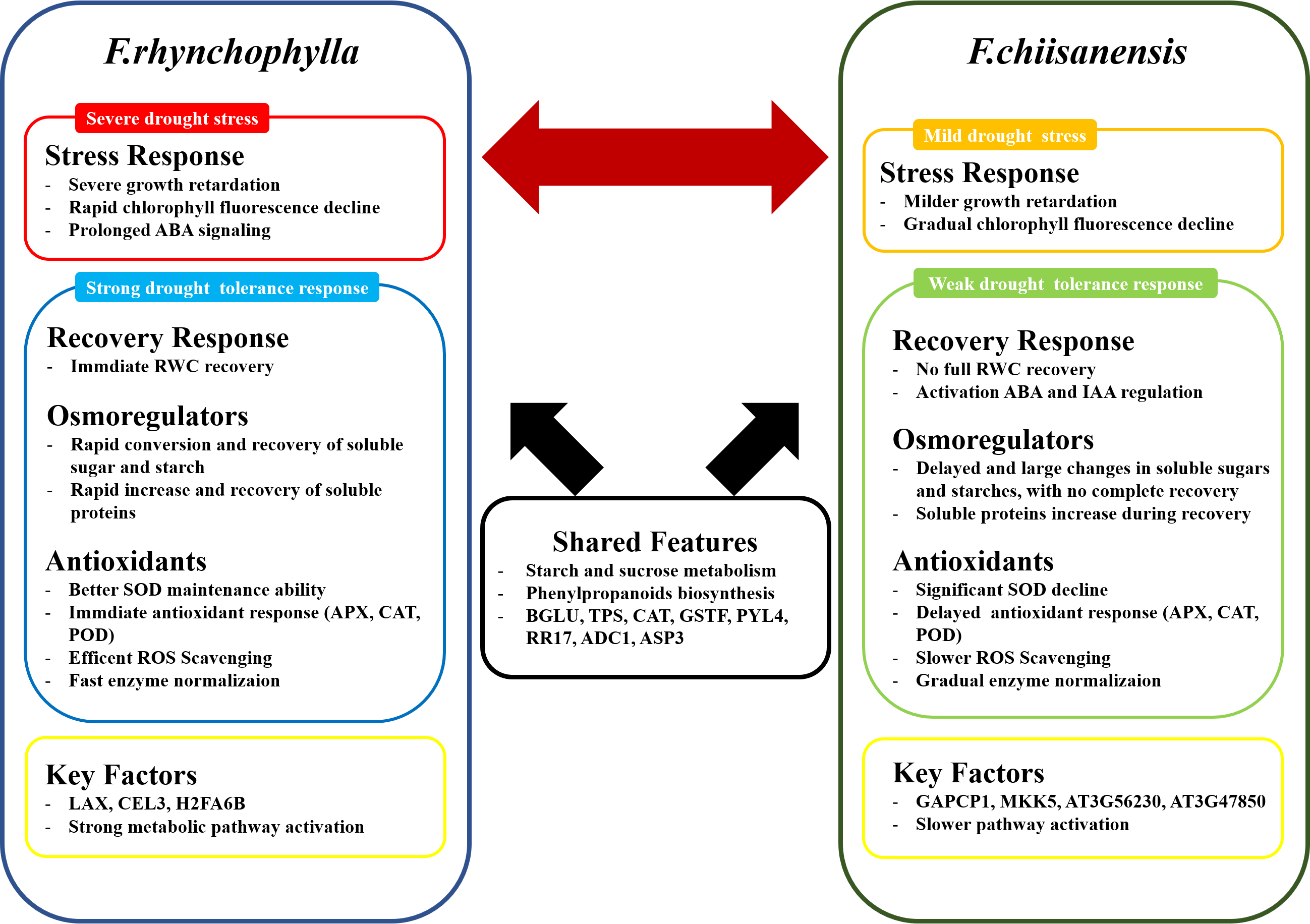

Supplement: Supplementary file 3 — Supplementary Material 3 [file 12870_2025_6372_MOESM3_ESM.docx]
